# A comprehensive review of the application of probiotics and postbiotics in oral health

**DOI:** 10.3389/fcimb.2023.1120995

**Published:** 2023-03-08

**Authors:** Aziz Homayouni Rad, Hadi Pourjafar, Esmaeel Mirzakhani

**Affiliations:** ^1^ Department of Food Science and Technology, Faculty of Nutrition & Food Sciences, Tabriz University of Medical Sciences, Tabriz, Iran; ^2^ Dietary Supplements and Probiotic Research Center, Alborz University of Medical Sciences, Karaj, Iran

**Keywords:** dental caries, tooth decay, microbiota, microbiome, probiotic, postbiotic, functional foods

## Abstract

Oral diseases are among the most common diseases around the world that people usually suffer from during their lifetime. Tooth decay is a multifactorial disease, and the composition of oral microbiota is a critical factor in its development. Also, *Streptococcus mutans* is considered the most important caries-causing species. It is expected that probiotics, as they adjust the intestinal microbiota and reduce the number of pathogenic bacteria in the human intestine, can exert their health-giving effects, especially the anti-pathogenic effect, in the oral cavity, which is part of the human gastrointestinal tract. Therefore, numerous *in vitro* and *in vivo* studies have been conducted on the role of probiotics in the prevention of tooth decay. In this review, while investigating the effect of different strains of probiotics *Lactobacillus* and *Bifidobacteria* on oral diseases, including dental caries, candida yeast infections, periodontal diseases, and halitosis, we have also discussed postbiotics as novel non-living biological compounds derived from probiotics.

## 1 Introduction

**Figure 1:**
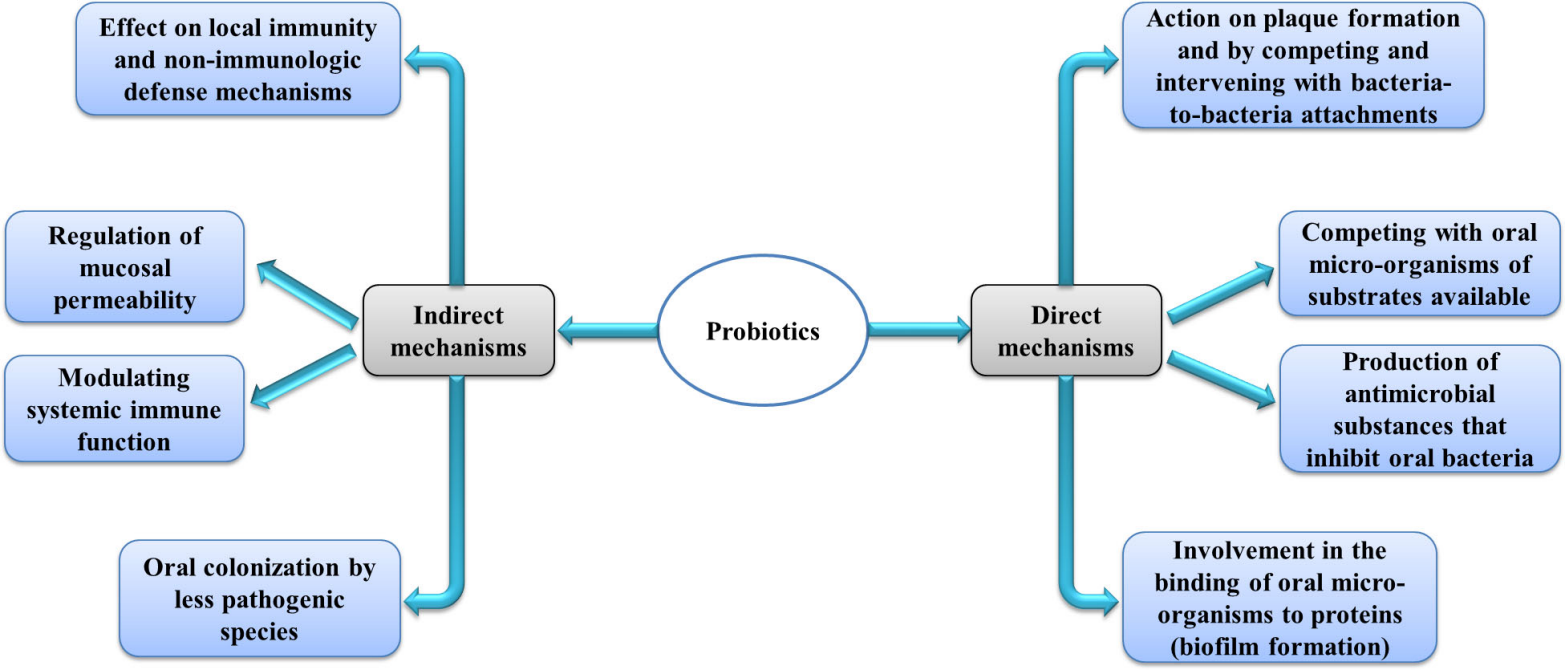
Possible mechanisms of probiotic in the oral cavity.

The World Health Organization estimates that about half of people suffer from oral diseases. About 2.4 billion people have permanent teeth decay and, 532 million children are also affected by primary teeth decay (Collaborators et al., 2020). Oral health care is expensive, and in high-income countries, about 5% of the total health costs are spent on it. In other countries, the required costs are higher than the capacity (Collaborators et al., 2020). Tooth decay is a disease related to several different factors. Factors such as a cariogenic diet, poor oral health, high counts of cariogenic bacteria, dental plaque, inadequate saliva flow, and lack of sufficient fluoride exposure are among the environmental risk factors that cause tooth decay (Petersen, 2003; Selwitz et al., 2007; Laleman et al., 2014; Iheozor-Ejiofor et al., 2015; Laleman and Teughels, 2015). As a general fact confirmed by various studies, the composition of oral microbiota is closely associated with oral health, and its disruption is an important step in oral disease (Zainab et al., 2016). *S. mutans*, one of the bacteria in the oral microflora, is known as the basic cause of dental decay and one of the biofilm-forming bacteria (Abd and Ali, 2016). Many clinical studies conducted on different people in the community have reported the correlation between *S. mutans* levels and caries (Loesche, 1986; Tanzer et al., 2001). It should be noted that the association between *S. mutans* levels and the occurrence of caries in people is different for various reasons, including genetic factors in the etiopathogenesis of tooth decay, and certain patients may be more vulnerable or resistant to caries (Werneck et al., 2010; Renuka et al., 2013). Although the intervention of various factors is effective in causing tooth decay, microbiological factors are the main factor. Therefore, it is believed that bacteriotherapy can be an effective way to prevent oral disease (Flichy Fernández et al., 2010; Laleman et al., 2014; Laleman and Teughels, 2015; Gruner et al., 2016). Since the oral cavity is the first part of the gastrointestinal system, it is logical that probiotics exert their effects on the oral and affect its microbiota just like the gut (Burton et al., 2013). probiotics are live microorganisms that, if consumed in enough amounts, are advantageous to host health (Walker and Buckley, 2006). *In vitro* studies have shown the advantageous effects of different strains of probiotics against oral pathogens (Dierksen et al., 2007; Haukioja et al., 2008; Hedberg et al., 2008; Twetman et al., 2009a; Lee et al., 2011; Lee and Kim, 2014; Han et al., 2019). *In vivo* studies have also mentioned the effects of probiotics in the prevention or cure of periodontal diseases and tooth decay. However, some trials have reported conflicting results (Gruner et al., 2016; Jayaram et al., 2016). The possible mechanisms of probiotics’ action on oral health are the same mechanisms mentioned in gastrointestinal studies (Meurman, 2005) (**Figure 1**). Evidence suggests that part of the antimicrobial effects of probiotics is associated with the substances they produce, including bacteriocins, organic acids, fatty acids, and hydrogen peroxide (Miles, 2007; Cheng et al., 2021). Considering some of the challenges and limitations that exist for using probiotics in food products, the use of metabolites or their non-living components (postbiotics) can be a new and appropriate solution (Rad et al., 2021b). Among the challenges that are related to the use of probiotics and can be solved by postbiotics is the creation of clinical problems, especially in people with a weakened immune system (Sartor, 2016; Homayouni Rad et al., 2020b), the probability of transmission of resistance genes to pathogenic microorganisms and the emergence of antibiotic resistance (Mathur and Singh, 2005; Homayouni-Rad et al., 2020a), and the need for high costs to provide an ongoing cold chain from production to consumption (Sawada et al., 2016). Accordingly, in this review, while examining the effects of different strains of probiotics on oral and dental health, we will also look at the impact of postbiotics.

## 2 Microbiome and oral health

**Figure 2:**
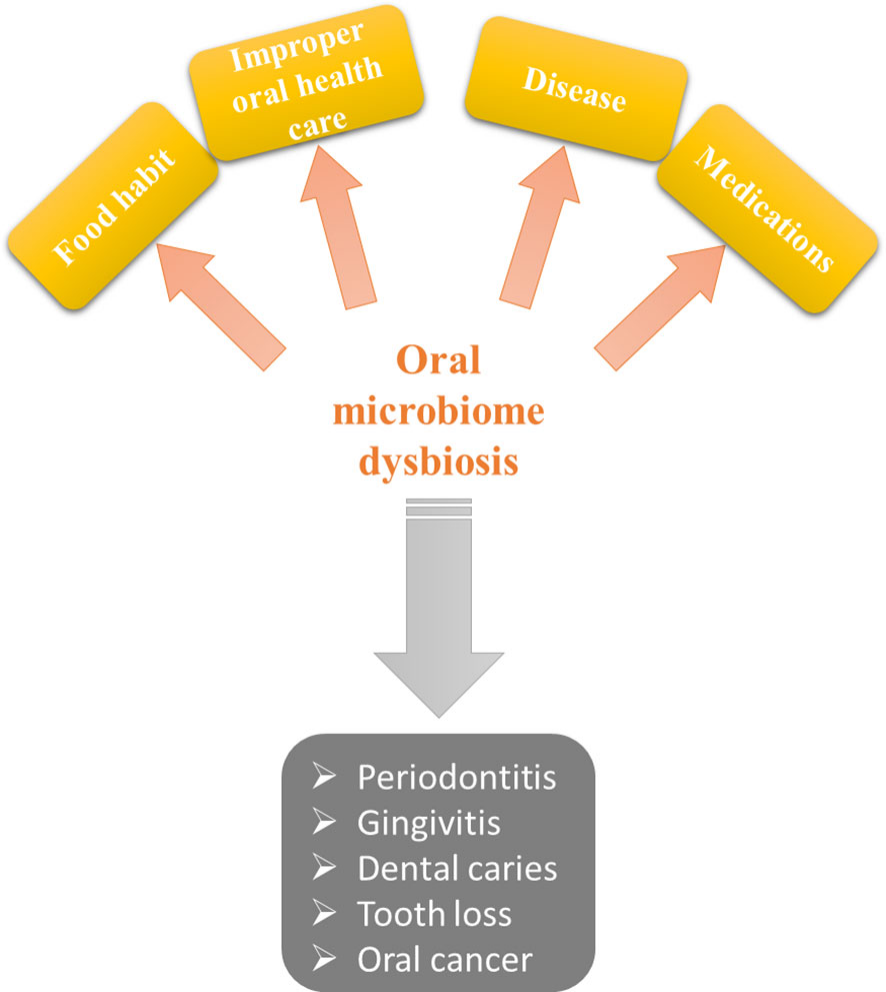
Pathogenicity of the oral microbiome in dysbiosis.

Microbes live in different parts of the body, and the ratio of the count of cells in the human body to the count of microbes is about 1:10 (Hillman et al., 2017). Advances in biological technologies have led to many studies to understand better the composition and impact of microbiota on human diseases (Gill et al., 2006). There are four main locales of microbial colonization in the body: the oral, intestines, skin, and vagina (Relman and Falkow, 2001); therefore, the oral cavity is one of the largest microbial sites, and the microbiome is stored on the teeth, tongue, soft and hard palates, gingival sulcus, and tonsils, and is an important factor in health and diseases related to the oral and teeth (Aas et al., 2005; Paster et al., 2006) (**Figure 2**). Using whole metagenome sequencing methods, it has been determined that bacteria such as *Actinobacteria*, *Proteobacteria*, *Fusobacteria*, *Bacteroidetes*, and *Firmicutes* constitute about 80-95% of the total oral microbiome (Bik et al., 2010; Ahn et al., 2011). The most prevalent species identified in the oral cavity using different methods including *Actinomyces*, *Prevotella*, *Streptococcus*, *Fusobacterium*, *Leptotrichia*, *Veillonella, Rothia*, *Corynebacterium*, *Capnocytophaga*, *Selenomonas*, *Treponema*, *Haemophilus*, and TM7 Genes 1 and 5 (Liu et al., 2012). When oral homeostasis is disrupted, and the dominance of good bacteria is lost, the conditions for dysbiosis and the growth of a diverse population of pathogenic bacteria with parasitic lifestyles are created. Liu et al. (2012) showed that dysbiosis occurs before the clinical symptoms of periodontal disease are evident, so examining the balance of oral microbiota can help as a tool for the initial detection of periodontitis. Also, in disease conditions, oral bacterial diversity decreases, and fewer bacterial genera predominate. For example, Gomar-Vercher et al. (2014), after collecting saliva samples from 12-year-old children, found that the two bacteria, *Porphyromonas* and *Provetella*, show an increasing percentage in children with caries compared to healthy people, and as the severity of the disease increases, bacterial diversity decreases. Two factors play a role in tooth decay: the immune system and diet. The oral microbiota creates an acidic environment on the teeth as a result of a frequent carbohydrate diet, and in this situation, acid-tolerant and acidogenic bacteria probably cause demineralization and cause tooth decay (Tanner et al., 2018). The study by Palmer et al. (2010) also showed that caries risk assessment often involves diet and bacteria and showed a correlation between the detection and abundance of *S. mutans* with the existence of caries. A study of 39 healthy and 51 caries people showed that the bacterial profiles of intact enamel in healthy people were significantly different from the bacterial profiles of intact enamel in sick people (Aas et al., 2008). Species like *Staphylococcus intermedius*, *Eubacterium saburreum* clone GT038, *Kingella oralis*, *Streptococcus cristatus*, and *Gemella morbillorum* were found at a high level in healthy people. They were at significantly decreased levels in the healthy enamel of diseased people in permanent teeth. Other health-related species in permanent teeth, like *Streptococcus* sp. group H6, *Streptococcus* sp. clone CH016, *Eubacterium* sp. clone EI074, *Campylobacter showae*, *Capnocytophaga sputigena*, *Leptotrichia* sp. clone DT031, and *Fusobacterium nucleatum* subsp. *polymorphum* were found at decreased levels in plaque from white spot or dentin lesions of diseased people compared to the levels detected in healthy people (Aas et al., 2008). On the other, *S. mutans*, *Streptococcus salivarius*, *Lactobacillus fermentum*, *L. gasseri*, *Prevotella* clone AO036, *Propionibacterium* FMA5, and *Atopobium genomospecies* C1 were found in the teeth of healthy subjects at low levels, while in dentin cavities and deep-dentin cavities were found at high levels, so caries-associated species were considered (Aas et al., 2008). In The study by Johansson et al. (2016), a clear correlation was observed between the frequency of *S. mutans* and caries. In this study, Romanian teenagers with an average age of 14.4 and a history of minimal dental care were found to have abundant dental plaque and a mean decayed, missing filling surface (DMFS) score of 20.1. In contrast, Swedish teenagers with an average age of 17 and a mean DMFS of 7.5 had regular dental care and also had a minimum observable dental plaque. On top of having more caries experience, Romanian teenagers showed a higher abundance of *S. mutans* diagnoses than Swedish ones (85% compared to 50%). On the other hand, there is evidence of microbiotas associated with non-*mutans* caries and not identifying *S. mutans* in about 10-15% of people with active caries (Hardie et al., 1977; Boyar et al., 1989; Van Houte, 1994; Beighton, 2005; Aas et al., 2008). These data suggest that potential acid-producing species are involved in caries, some of which have not yet been cultivated (Fitzgerald and Keyes, 1960). For example, *Scardovia wiggsiae* was detected in about 40% of children with active caries who did not have *S. mutans* (Tanner et al., 2011). Therefore, this species may be one of the important factors of caries in the absence of *S. mutans*. In a study by Hughes et al. (2012), different parts of the microbiota were cultured in rich blood containing agar and in an acid isolation medium with low pH. The greatest difference between the microbial population of children without tooth decay and ECC (Early childhood caries) children was in the acid isolation medium. The results of this study showed that there was a significant difference between health and caries in the acid-resistant population. Acid-resistant species included were the *S. mutans*, *Actinomyces odontolyticus*, and several having no name *Actinomyces* spp., *Bifidobacterium*, *Scardovia*, and *Lactobacillus* species. Also, two species related to caries, i.e., *S. mutans* and *S. wiggsiae*, had a significant difference between disease and health conditions in both types of bacterial isolation in acid or blood agar conditions (Hughes et al., 2012). In a study, researchers cultivated several new species of *Actinomyces* that were resistant to acidic conditions. Therefore, the potential for acid production at a low pH was considered to Discover the caries potential (Van Houte, 1994). For a long time, *Actinomyces* have been known as part of the caries microbiome and have been a significant group in the induction and development of caries (Takahashi and Nyvad, 2011). Several studies have also associated *Veillonella* spp. with dental decay. Bradshaw and Marsh (1998) reported that *Veillonella* was the most populous organism, especially in low pH after all glucose-pulsing regimens. Noorda et al. (1988) reported that acid production was higher in bacterial plaques mixed with *V. alcalescens* and *S. mutans* than plaques that consist of only one of these two species. Also, Mikx and van der Hoeven (1975) confirmed an active relationship between acid-producing bacteria and *Veillonella* species. On the other hand, *Veillonella* spp. may be consequential for acid-producing bacteria by reducing nitrate (Doel et al., 2005). Silva Mendez et al. (1999) reported that low concentrations of nitrite and pH levels of less than 7 (0.2 mM) killed *S. mutans*. These findings indicate that nitrite in saliva affects the growth and survival of cariogenic bacteria. Nadkarni et al. (2004) and Chhour et al. (2005) found that *Prevotella*-like and *Prevotella* bacteria master the varied polymicrobial community in some cases of caries, which suggests that *Prevotella* play a role in the development of dental decay. In addition, co-aggregation between six species of *S. constellatus* ND10-13A, *S. bovis* II/2 ND2-2, *L. acidophilus* ND7-2A, *S. sanguinis* II ND7-3, *C. sputigena* ND2-12A, *P. intermedia* ND8-9A, and *F. nucleatum* NT6-6A, showed that these strains can co-aggregate with many oral bacteria and while forming dental plaque and bringing it to maturity, they will also control the composition of the microbial community (Kolenbrander et al., 1983; Kolenbrander, 2000). Evidence suggests that a wide range of bacterial strains are involved in tooth decay. In the study conducted by Chhour et al. (2005), also it was stated that there were 75 species or phylotypes in 10 caries lesions. Up to 31 species have been reported separately in each sample. Various species of *Lactobacilli* make up about 50% of the species, and about 15% are *Prevotellae*. Other species with high affluence are *F. nucleatum*, *Pseudoramibacter alactolyticus*, *Dialister* spp., *Selenomonas* spp., *Eubacterium* spp., *Olsenella* spp., *Bifidobacterium* spp., members of the *Lachnospiraceae* family, and *Propionibacterium* sp. (Chhour et al., 2005). In more studies, it was stated that *Lactobacillus* and *Mutans* spp. are dominant in advanced caries (Munson et al., 2004; Corby et al., 2005). In a study, it was stated that the number of *S. mutans* decreased after the cure compared to before the cure (Hughes et al., 2012). While, in another study, it was indicated that the treatment was associated with a change in the microbial profile, but this change was not significant for *S. mutans* (Tanner et al., 2011). The object of the treatment is to keep the tooth structure and intercept its further decay (Khushbu and Satyam, 2016), but since there is evidence that dental decay at first is a reversible disease that has a multifactorial cause, dentists are no longer seeking to use interventional treatment at a specific starting point of the appearance and severity of the disease. Nowadays, it is quite explicit that the restorative treatment of dental caries alone does not treat caries, and the caries process should be controlled with the patient’s participation throughout his life (Pitts, 2004). Also, considering that the treatment of dental diseases is not possible for all members of society due to the need to spend a lot of money and time, prevention is a more economical way. Therefore, personal hygiene and diet modification should be considered (Khushbu and Satyam, 2016). It has been said about children that their oral microbiota originates from the oral microbiota of the child’s main caregiver, the mother. Therefore, it is very important to focus preventive and therapeutic measures on child caregivers to prevent tooth decay in children (Tanner et al., 2002).

## 3 *Lactobacillus* bacteria and oral health

Tooth decay is a local demineralization of the hard tissues of the crown and root surface of the tooth, which happens in a bacterial accumulation that adheres to the tooth surfaces, called dental plaque, which is made of a gelatinous substance (Gupta and Gupta, 2015). *S. mutans* has been introduced as the main species of caries-causing bacteria due to its strong acid production and high tolerance to acidic conditions (Sales-Campos et al., 2019; Hasslöf and Stecksén-Blicks, 2020). Research has shown that the number of *S. mutans* in the saliva of people without caries is normally between 10^4^ and 10^5^ CFU/mL (Deepti et al., 2008), But at 10^6^ CFU/mL, the risk of caries increases significantly (Klock and Krasse, 1979). In this regard, 10^5^ CFU/mL has been considered the threshold of caries in clinical studies (Caglar et al., 2008b; Ghasemi et al., 2017; Bafna et al., 2018).

Probiotic bacteria may cause chemical and physical alterations in the microbial flora of people’s oral cavity (Teughels et al., 2007). Theoretically, probiotics have stronger adhesion to oral tissues than pathogens and can compete for adhesive surfaces. This leads to bacterial aggregation and co-aggregation and the formation of a new biofilm (Twetman, 2012; Piwat et al., 2015; Takahashi, 2015; Morales et al., 2016). Probiotics compete with the oral microbial flora and pathogens for adhesion sites, nutrients, and growth factors, thus protecting oral health. These bacteria aggregate after sticking to the oral cavity and prevent the adhesion of pathogenic bacteria through the production of antimicrobial components like acids, bacteriocins, and peroxides. Therefore, probiotic bacteria may prohibit the growth of caries-causing bacteria and periodontal diseases, create an immune response against pathogens, and prevent oral tissue destruction and inflammation in the oral cavity (Sanders, 1969; Yasui et al., 1999; Roberts and Darveau, 2002; Wilson, 2005; Twetman, 2012; Twetman and Keller, 2012; Devine et al., 2015; Laleman et al., 2015; Gruner et al., 2016; Morales et al., 2016). Various researchers have surveyed the effect of the consumption of probiotics on tooth decay. For example, (Jindal et al., 2011); Wattanarat et al. (2015), concluded that the use of probiotics could decrease the count of *S. mutans* and thus have a prohibitory effect on tooth decay. Nadelman et al. (2018) concluded during a meta-analysis that probiotics, including *Lactobacillus*, *Bifidobacterium*, and *Streptococcus*, could significantly reduce the count of *S. mutans* compared to control groups and prevent tooth decay. The results of this meta-analysis are consistent with systematic reviews that showed a significant decrease in the count of *S. mutans* after probiotic consumption (Cagetti et al., 2013; Laleman et al., 2014; Gruner et al., 2016).

Strains such as *Bifidobacterium* spp. and *L. rhamnosus*, *L. reuteri*, and *L. casei* have all shown the capability to change the colonization of carious bacteria, which can prevent tooth decay (Meurman and Stamatova, 2007). Kõll‐Klais et al. (2005b) examined *Lactobacillus* strains in their research and reported that 69% of the strains could inhibit the growth of *S. mutans*, and 82% of the strains can inhibit the growth of *Porphyromonas gingivalis*. Both *L. salivarius* and *L. fermentum* have been found to have antagonistic activity against the growth of *S. mutans* (Strahinic et al., 2007). Also, *L. salivarius* TI 2711 isolated from a healthy human has shown an inhibitory effect on *P. intermedia*, *P. nigrescens*, and *P. gingivalis* after co-cultivation (Ishikawa et al., 2003). In a study by Sookkhee et al. (2001), the ability to inhibit the growth of various oral pathogens by 3790 lactic acid bacteria (LAB) was investigated, and it was stated that *L. rhamnosus* and *L. paracasei* have a strong antibacterial effect on several oral pathogens. Considering the digestive benefits of *Lactobacilli* and the fact that the *L. rhamnosus* GG strain is not cariogenic due to its inability to consume sucrose or lactose (Homofermentative *Lactobacilli*), Meurman et al. (1995) became the first researcher to show the inhibitory effect of this bacterium on a cariogenic pathogen. Therefore, it is expected that this bacterium has been conducive to oral health (Stamatova et al., 2009). In this regard, it has been stated that *L. rhamnosus* GG ATCC 53103 produces a growth-prohibitory compound against *Streptococcus sobrinus* and is suggested to decrease the risk of caries (Haukioja et al., 2006). *Lactococcus lactis* has been introduced as another effective strain of *Lactobacilli* in eliminating the colonization of pathogenic oral bacteria and modulating the oral microflora. This bacterium has shown the ability to eliminate the colonization of bacteria such as *Actinomyces*, *V. dispar*, *S. oralis*, and cariogenic *S. sobrinus* (Comelli et al., 2002).

On the other hand, some studies have claimed that *Lactobacillus* themselves are correlated to the initiation and development of caries (Chuang et al., 2011; Sidhu et al., 2015). During their metabolism, *Lactobacilli* produce acids that may increase the risk of caries (Huang et al., 2015). Shimada et al. (2015) found that the number of *Lactobacilli* was significantly higher in children who had tooth decay than in children without decay. Therefore, they concluded that the number of *Lactobacilli* in the oral cavity is associated with the progression of caries. In other studies, evidence suggests that *Lactobacilli* are more correlated with caries development than with initiation of caries and play a momentous impress in the development of tooth decay (Edwardsson, 1974; Maltz et al., 2002; Karpiński and Szkaradkiewicz, 2013; Caufield et al., 2015). Also, it has been reported that the count of *Lactobacilli* after probiotic consumption was not significantly different between the control and experimental groups (Çaglar et al., 2005; Çaglar et al., 2006; Caglar et al., 2007; Caglar et al., 2008b; Petersson et al., 2011; Singh et al., 2011; Cildir et al., 2012; Mortazavi and Akhlaghi, 2012). In a study, it was stated that the count of *Lactobacilli* decreased in one of the two probiotic groups after oral probiotic consumption (Cogulu et al., 2010). In contrast, it was noted in two studies that the count of *Lactobacilli* increased significantly (Aminabadi et al., 2011; Keller and Twetman, 2012). Therefore, according to the reports of most studies, it can be claimed that the consumption of probiotics does not cause the initiation of tooth decay. In this regard, it was reported in an intervention study that the consumption of *Lactobacilli* by children decreased the risk of tooth decay and primary caries in them (Reddy et al., 2011). Until now, the effect of the consumption of different strains of probiotics on tooth decay has been investigated in several studies [**Table 1**]. Regarding the literature investigation, most studies stated that the consumption of probiotics efficiently prevents tooth decay development, and they advise a controlled consumption of probiotics to reach advantageous effects.

**Table 1:** Human *in vivo* studies on the effect of *Lactobacilli* on oral health.

The studied community	Number	Probiotic strain	Dose of probiotics (CFU/ml)	Carrier food	Study period	Result	Reference
Children (1-6 years)	571	*L. rhamnosus* GG	5-10×10^5^ (Three times a day, 200 ml each time, 5 days a week)	Milk	7 months	Reduce the risk of decay	(Hatakka et al., 2001)
Children (7-14 years)	150	*L.rhamnosus, B.lonum*, *Saccharomyces cerevisiae*	1.25×10^9^/20ml	Water (20 ml)	14 days	A statistically significant decrease (p<0.001) in the count of *S. mutans* in saliva was recorded after 14 days of probiotic ingestion	(Jindal et al., 2011)
Children (6-12 years)	60	*L.reuteri*	2×10^8^	Lozenge	28 days	A significant reduction in the count of *S. mutans* and *Lactobacilli* in saliva and decreasing the risk of tooth decay	(Cannon et al., 2013)
Adults (21-24 years)	120	*L.reuteri* ATCC 55730	–	Group A: water (200 ml) Group C: tablet (once a day)	3 weeks	Unlike *Lactobacilli*, a significant reduction was observed in salivary *S. mutans*	(Çaglar et al., 2006)
Adults (18-35 years)	74	*L. rhamnosus* LC705, *L. rhamnosus* GG	*L. rhamnosus* LC705 (1.2 × 10^7^) and *L. rhamnosus* GG (1.9 × 10^7^)	Cheese (5×15 g per day)	3 weeks	The results indicated that there was no significant difference between the groups in the number of *S. mutans* after the intervention, but a significant decrease was observed in the intervention group compared to the control group during the post-cure period (P=0.05)	(Ahola et al., 2002)
Adults (20-35 years)	28	*L. casei* shirota	10^8^	Fermented milk (65 ml daily)	4 weeks	Reduction of biomarkers associated with gingival inflammation and reduction of gingival crevicular fluid volume and bleeding in probing scores	(Slawik et al., 2011)
Adults (20-26 years)	78	*L. paracasei* GMNL-33	3×10^8^	Tablet (thrice a day)	2 weeks	Significant reduction in the count of caries *S. mutans*. A 2-week oral administration period for *L. paracasei* GMNL-33 may be required for the probiotic to become effective	(Chuang et al., 2011)
Adults (18-25 years)	40	*L. paracasei* SD1	7.5×10^8^	Reconstituted milk powder (10 g in 50 ml water)	4 weeks	The results showed that short-term daily use of *L. paracasei* SD1 reduces the number of oral *S. mutans*	(Teanpaisan and Piwat, 2014)
Adults (average of 25 years)	64	*L. salivarius* WB21, *L. salivarius* TI 2711	*L. salivarius* WB21: 6.7 × 10^8^ CFU/tablet, *L. salivarius* TI 2711: 2.8 × 10^8^ CFU/tablet	Tablet	2 weeks	In both *L. salivarius* WB21 and TI 2711 groups, the count of *S. mutans* decreased and the count of *Lactobacilli* increased.	(Nishihara et al., 2014)
older adults (58-84 years)	160	*L. rhamnosus* LB21	10^7^	Milk (200 ml daily)	15 months	If milk with fluoride and/or probiotics is consumed daily, the lesions of soft and leathery primary root caries may be reversed in older people	(Petersson et al., 2011)
Children (1-5 years)	248	*L. rhamnosus* LB21	10^7^	Milk (150 ml with 2.5 mg fluoride per liter)	21 months	The results showed that daily use of probiotics and fluoride by preschool children reduces the incidence of caries	(Stecksén-Blicks et al., 2009)
Children (2-3 years)	261	*L. rhamnosus* SP1	10^7^	Milk (150 ml)	40 weeks	Reducing the severity and progression of caries in children compared to the control group	(Kaye, 2017)
Children (7-12 years)	40	*L. reuteri* (ATCC 55730 and ATCC PTA 5282)	–	Chewing gum (three a day)	3 weeks	In addition to the decrease in the count of salivary *s. mutans* after the intervention, a significant reduction in plaque and gingival scores was also observed	(Kaur et al., 2018)
Children (12-14 years)	122	*L. paracasei* SD1	10^7^	Milk powder (5g/day)	6 months	After completing the study, fewer caries lesions were detected in the probiotic-consuming group. Also, in the group with a high risk of caries, a significant reduction in new caries was detected (4.5 times), while no such decrease was observed in the group with a low risk of caries	(Teanpaisan et al., 2015)
Children (1.5-5 years)	124	*L. paracasei* SD1	10^7^	Milk powder (5g/day)	3 month	The use of milk powder containing *L. paracasei* SD1 as a safe strain for young children reduced the levels of salivary *S. mutans* and also delayed the development of new caries.	(Pahumunto et al., 2018)
Children (12-14 years)	40	*L. paracasei* SD1	–	Milk powder (5g/day)	6 months	*L. paracasei* SD1 able to control the level of *S. mutans* and stimulate sIgA	(Pahumunto et al., 2019)
Infants	171	*L. paracasei* F19	10^8^	Cereals	9 months	Early intervention with LF19 did not affect the prevalence of dental caries, *S. mutans*, or *Lactobacilli.*	(Hasslöf et al., 2013)
Children (13-14 years)	201	*L. rhamnosus* SD11	5×10^10^ (Fermented milk) and 7.5×10^7^ (condensed milk powder)	Fermented milk and condensed milk powder	9 months	The consumption of probiotics by children caused the number of *S. mutants*, the percentage of caries progress, and caries lesions to decrease significantly.	(Piwat et al., 2019)
Children (average of 16 years)		*L. reuteri* (ATCC PTA 5289 and DSM 17938)	10^9^	Lozenge	17 month	No significant change was observed between the treatment and placebo groups in the incidence of white spot lesions, as well as the count of *S. mutans* and *Lactobacilli*.	(Gizani et al., 2016)
Children (6-10 years)	25	*L. rhamnosus* GG ATCC 53103	–	Yogurt	2 weeks	Thirty days after consuming yogurt, a significant increase in the buffer capacity of saliva was observed. Also, the number of *S. mutans* decreased significantly, but the number of *Lactobacillus* did not change significantly.	(Glavina et al., 2012)
Children (12-15 years)	40	*L. rhamnosus* hct 70	2.34×10^9^ CFU/daily	Milk (150 ml)	3 weeks	Probiotic consumption reduced the count of *S. mutans* immediately after the end of the intervention and also after 3 weeks.	(Juneja and Kakade, 2012)
Children (6-8 years)	31	*L. casei* Shirota	–	Milk (60 ml)	10 days	A significant decrease (p=0.003) in the number of *S. mutans* colonies was observed in the group consuming probiotics.	(Yadav et al., 2014)
Adults (average of 25 years)	13	*L. reuteri* (PTA 5289 and SD2112) or *L. rhamnosus* GG	2×10^6^/tablet L. reuteri, 1.96×10^6^/tablet L. rhamnosus	Tablet	2 weeks	The number of *S. mutants* remained unchanged, and no change in the acidogenicity of plaque was observed with probiotic consumption. In the *L. reuteri* group, the number of people with *lactobacillus* in the plaque increased (p=0.011), unlike the *L. rhamnosus* group.	(Marttinen et al., 2012)
Adults (average of 26 years)	18	*L. reuteri* (ATCC PTA 5289 and DSM 17938)	10^8^ of each strain	Lozenge	2 weeks	The number of salivary *S. mutans*, unlike *Lactobacilli* (p<0.05), in the experimental group did not show any significant changes during the intervention.	(Keller and Twetman, 2012)
Adults (19-35 years)	62	*L. reuteri* (ATCC PTA 5289 and DSM 17938)	10^8^ of each strain	Lozenge	6 weeks	It seems that daily use of *L. reuteri* cannot delay the regrowth of *S. mutans* after thorough oral disinfection using chlorhexidine	(Keller et al., 2012a)
Adults (21-24)	80	*L. reuteri* (ATCC PTA 5289 and ATCC 55730)	1 × 10^8^ CFU/gum ATCC 55730 and 1 × 10^8^ CFU/gum ATCC PTA 5289	Chewing gum	3 weeks	Unlike salivary *Lactobacilli*, the count of *S. mutans* in the probiotic consumption group showed a significant decrease compared to the baseline level after the intervention	(Caglar et al., 2007)
Adults (average of 20 years)	20	*L. reuteri* (ATCC PTA 5289 and ATCC 55730)	1.1×10^8^	Lozenge (once daily)	10 days	In the probiotic consumer group, the number of salivary *S. mutans* was significantly decreased (P < 0.05)	(Caglar et al., 2008a)
Infants	113	*L. reuteri* ATCC 55730	10^8^	Oil derived from breast milk	13 months	Daily consumption of *L. reuteri* from birth to one year has reduced the prevalence of caries and gingivitis score in primary dentition at the age of nine	(Stensson et al., 2014)
Children (4-12 years)	19	*L. reuteri* (ATCC PTA 5289 and DSM 17938)	≥10^8^ CFU/5 drops	Drops (5 per day)	25 days	There was no significant decrease in the number of salivary *S. mutans* and *Lactobacillus* after 25 days of probiotic consumption	(Cildir et al., 2012)
Adolescent (12-17 years)	36	*L. reuteri* (ATCC PTA 5289 and DSM 17938)	10^8^ of each strain	Tablet	3 months	There was no significant change in the number of *S. mutans* and *Lactobacillus* in the treatment group. It also seems that this probiotic does not affect adolescent decay lesions	(Keller et al., 2014)
Children (6-8 years)	191	*L. brevis CD2*	2×10^9^	Lozenge	6 weeks	A significant decrease was observed in the count of salivary *S. mutans*, plaque acidogenicity, and bleeding on probing	(Campus et al., 2014)
Adults	42	*L. reuteri* (ATCC PTA 5289 and ATCC 55730)	10^8^ CFU/gum	Chewing gum (One or two a day)	2 weeks	After consuming these strains, it was found that the volume of gingival crevicular fluid and bleeding in the probe improved significantly (p<0.05)	(Twetman et al., 2009b)
Adults (20 years)	40	*L. reuteri*	–	Yogurt (95 g once daily)	2 weeks	*L. reuteri* consumption significantly decreased the number of *S. mutans* compared to placebo (yogurt without probiotics)	(Nikawa et al., 2004)
The elderly	276	*L. lactis* and *Lactobacillus helveticus*	10^7^ of each strain	Cheese (50 g)	16 weeks	Probiotics can control hyposalivation and oral *Candida* in older people.	(Hatakka et al., 2007)
Adults (average of 20 years)	60	–	–	Curd (200 mg) and toothpaste (twice daily)	30 days	The use of probiotics significantly reduces the count of *S. mutans* in the plaque formed around the bracket in orthodontic patients.	(Jose et al., 2013)
Adults (22-32 years)	22	*Saccharomyces cerevisiae, L. casei* subsp. *Pseudo plantarum*	–	Kefir drink (100 ml per day)	2 weeks	Consuming a probiotic Kefir drink can inhibit the number of salivary *S. mutans*. It may also be selected as an alternative to fluoride washing.	(Ghasempour et al., 2014)
Adults (20-25)	60	*L. acidophilus*	–	Curd (100ml)	7 days	In the short term, probiotic consumption reduced the number of salivary *S. mutans* and increased salivary pH.	(SrivaStava et al., 2016)
Adults	57	*L. salivarius* TI 2711 (LS 1)	2×10^7^	Tablet	4 or 8 weeks	With probiotic consumption, the number of whole bacteria, *S. mutans*, and *Lactobacilli* did not change, but the number of *P. intermedia*, *P. nigrescens*, and *P. gingivalis* decreased.	(Ishikawa et al., 2003)

## 4 *Bifidobacteria* and oral health

Interest in using probiotic *Bifidobacteria* to prevent and treat oral microbial diseases is increasing. Nevertheless, the results reported from clinical studies aimed at investigating the effect of *Bifidobacteria* on oral microbiota are conflicting and controversial. Some studies have reported that *Bifidobacteria* have anti-caries effects, and others have published the opposite results. Few studies have surveyed the effect of *Bifidobacteria* on *S. mutans*, and some of these studies have also surveyed the effects of *Bifidobacteria* along with *Lactobacilli*. In the meta-analysis conducted by Gruner et al. (2016), it was shown that *Bifidobacterium* does not effectively reduce the count of *Lactobacilli* in saliva. Still, it has a significant reduction effect on the count of *S. mutans*. Also, studies that used supplements of *Bifidobacterium* and *Lactobacillus* spp., reported a reduction in the count of *S. mutans* in the oral cavity (Mahantesha et al., 2015; Ghasemi et al., 2017). Similar results have been reported in people who did not use fluoride (Ghasemi et al., 2017). While in some studies, the use of *Bifidobacterium animalis* BB-12 and other species of *Bifidobacterium* genus in people who did not use fluoride failed to show its therapeutic effects on tooth decay (Pinto et al., 2014; Nozari et al., 2015). A meta-analysis was conducted to investigate the difference in the count of *S. mutans* in saliva before and after consuming *Bifidobacterium*. The pooled results of four studies did not show significant differences (Pinto et al., 2014; Nagarajappa et al., 2015; SrivaStava et al., 2016; Javid et al., 2020). In a study, it has been stated that *Bifidobacteria* did not have a significant effect on reducing the count of *S. mutans* but significantly reduced the number of *P. gingivalis* in the biofilm (Jäsberg et al., 2016). On the other hand, Valdez et al. (2016) showed that *Bifidobacterium* can make an acidic environment and enhance the formation of biofilm so that *Bifidobacterium* may have a cariogenic effect on teeth. Haukioja et al. (2008) stated that *Bifidobacterium* could cause tooth demineralization because of the production of organic acids. Also, Dual-species biofilms formed by *S. mutans* and *Bifidobacteria* produce more acid than *S. mutans* biofilm alone, and as a result, more pH drops will occur (de Matos et al., 2017). Zhai et al. (2009) investigated the distribution of *Bifidobacterium* in the oral cavity and the association between *Bifidobacterium* and tooth decay in children. In this clinical study, the results indicated that the rate of *Bifidobacterium* was 47.5% in patients with severe ECC and 0% in healthy people without caries. **Table 2** shows the results of clinical studies conducted to investigate the effect of *Bifidobacterium* on oral health. The number of studies that have used *Bifidobacteria* alone and separately from *Lactobacilli* is limited. This has made it challenging to decide on the effect of *Bifidobacteria* on the count of *S. mutans* and tooth decay. Therefore, in the future, more studies should be conducted with food products containing only *Bifidobacteria*.

**Table 2:** Human clinical studies on the effect of *Bifidobacteria* on oral health.

The studied community	Number	Probiotic strain	Dose of probiotics (CFU per ml/gram)	Carrier food	Study period	Result	Reference
Adults (18-22 years)	30	*Bifidobacteria*	10^6^	Chocobar ice cream (42 grams once a day)	18 days	The number of salivary S. mutans decreased in contrast to salivary *Lactobacilli* (p < 0.05).	(Nagarajappa et al., 2015)
Adults (21-24 years)	21	*Bifidobacterium* DN-173010	7×10^7^	Yogurt (200 grams daily)	2 weeks	decreased in the number of *S. mutans* in saliva, and no significant change in the number of *lactobacilli*.	(Çaglar et al., 2005)
Adults (mean age 20 years)	24	*Bifidobacterium* lactis Bb-12	–	Ice cream (53 grams daily)	10 days	The number of salivary *S. mutans* decreased in contrast to salivary *Lactobacilli* (p < 0.05).	(Caglar et al., 2008a)
Infants (1-2 months)	121	*B. animalis* subsp. lactis BB-12	10^10^ (daily dose)	Tablet (200-600 mg xylitol or sorbitol for control groups without probiotic)	2 years	It seems that the consumption of strain BB-12 in infants does not cause a change in the caries process until the age of 4 in the low caries population.	(Taipale et al., 2012; Taipale et al., 2013)
Children (12-14 years)	40	*B. lactis* Bb-12 ATCC27536, *L. acidophilus* La-5	10^6^ of each probiotic strains	Ice cream (54 gram daily)	10 days	A significant decrease in salivary *S. mutans* levels was observed (p=0.003), unlike *Lactobacilli* levels.	(Singh et al., 2011)
Children (6-12 years)	60	*B. lactis* Bb-12, *L. acidophilus* La-5	10^6^ of each probiotic strains	Ice cream (54 grams)	7 days	Within a week of consuming probiotic ice cream, a significant decrease in the number of *S. mutans* was observed (p<0.001).	(Ashwin et al., 2015)
Adults (18-30 years)	66	*B. lactis* Bb12	10^6^	Yogurt (300 grams daily)	2 weeks	Consumption of probiotics decreased the count of salivary *S. mutans* and *Lactobacilli* in the treatment group, both compared to the baseline level and compared to the control group.	(Javid et al., 2020)
Children (average age of 15 years)	26	*B. animalis* subsp. lactis DN-173010	–	Yogurt (200 gram daily)	2 weeks	After 2 weeks, the reduction in the count of *S. mutans* and *Lactobacilli* was not significant.	(Pinto et al., 2014)
Children (8-10 years)	52	*B. bifidum* DN- 173 010	10^10^	Fruit yogurt (110 gram daily)	2 weeks	No significant difference was observed in *S. mutans* levels in dental plaque before and after treatment.	(Caglar, 2014)
Young adults	60	*B. animalis* subsp. lactis BB-12, *L. rhamnosus* GG	2×10^9^ of each probiotic strains	Lozenge	4 weeks	Improvement of the periodontal condition was observed without affecting the oral microbiota after taking probiotics.	(Toiviainen et al., 2015)
Adults	70	*Bifidobacterium* lactis BB-12 and *L. acidophilus* La5	10^6^/100 grams	Yogurt (100 grams daily)	2 weeks	A decrease in the number of salivary *S. mutants* was observed after consuming yogurt containing *B. lactis* Bb12 and *L. acidophilus* La5.	(Bafna et al., 2018)
Children (3-4 years)	363	*L. rhamnosus*, *B. longum*	5×10^6^ L. rhamnosus, 3×10^6^ B. longum	Milk (200 ml daily)	9 months	The number of S. mutans in the probiotic consumption group was lower than in the control group, but this decrease was not significant (p=0.173).	(Villavicencio et al., 2018)
Children (3-5 years)	63	*B. longum*, *L. rhamnosus* GG	L. rhamnosus GG (7.5 × 10^5^) and B. longum (4.5 × 10^5^)	Milk (200 ml)	3 months (5 days a week)	Consumption of probiotic milk did not reduce the count of *S. mutans*, but it reduced the remineralization of tooth decay and saliva acidity.	(Angarita-Díaz et al., 2020)
Children (6-12 years)	49	*B. lactis*	10^6^	Yogurt (200g once daily)	2 weeks	use of probiotic yogurt containing *B. lactis* could not decrease the levels of *S. mutans* and *Lactobacilli* in saliva, while normal yogurt could decrease the count of *S. mutans* significantly	(Nozari et al., 2015)
Adults (19-27 years)	50	*B. bifidum* ATCC 29521, *L. acidophilus* ATCC 4356	1.5×10^8^	Yogurt (200 g daily)	3 weeks	On the first day, the seventh day, and one month after the intervention, the count of *S. mutans* was significantly lower than the baseline values (P < 0.05).	(Ghasemi et al., 2017)
Children (12-15)	33	*B. bifidum, L. acidophilus*, *B. lactis*, and *B. longum*,	–	Water (30 ml once daily)	3 weeks	Up to the second week, a significant decrease in the number of salivary *S. mutants* was observed (P<0.05).	(Yousuf et al., 2015)

## 5 Probiotics and *Candida* yeast infections

*Candida albicans* fungus is one of the prevalent infectious agents in the oral cavity. At older ages and in immunodeficiency conditions, the risk of yeast infections increases (Jiang et al., 2016). *Candida* has different species, and the species that are usually isolated from the oral cavity include *C. krusei*, *C. glabrata*, *C. tropicalis, C. parapsilosis*, and *C. albicans*. Increasing antifungal resistance is becoming a new issue worldwide, and new methods are needed to fight pathogenic fungi. Probiotic *Lactobacilli* have demonstrated different inhibitory effects against oral *Candida* during *in vitro* studies. Meanwhile, *L. Rhamnosus* GG has shown a strong prohibitory effect (Jiang et al., 2015). Haukioja et al. (2006) conducted a placebo-controlled study for the first time to examine the effect of probiotics on the abundance of oral *Candida*. Consumption of cheese containing *Propionibacterium freudenreichii* ssp. *shermanii* JS and *L. rhamnosus* GG probiotics decreased the abundance of *C. albicans* by 75% in the elderly. Yakult drink containing *B. breve* and *L. casei* increases salivary secretory protein levels and causes a significant decrease in *Candida* and non-*Candida* species in the oral cavity of the elderly (Mendonça et al., 2012). Also, the consumption of *L. reuteri* lozenge and probiotic cheese containing *L. rhamnosus* LC 705 and *L. rhamnosus* GG has reduced the number of oral yeasts correlated with caries (Ahola et al., 2002; Kraft-Bodi et al., 2015). During a randomized clinical trial that was conducted on 60 children, it was shown that a probiotic rinse for one week was as efficient as chlorhexidine digluconate 0.2% in decreasing the count of *C. albicans* (Mishra et al., 2016). It has also been reported that probiotic bacteria have an anti-biofilm effect against *C. albicans* (James et al., 2016; Matsubara et al., 2016) and against the ability to produce biofilms of two species of *C. albicans* and *S. mutans* (Krzyściak et al., 2017).According to these promising studies, it can be estimated that the expansion of research on the relationship between probiotics and oral yeast infections and the investigation of molecular means of probiotic activity may expand their potential applications in the future (Meurman and Stamatova, 2007).

## 6 Probiotics and periodontal diseases

Periodontal disease is inflammation of dental support tissues, which comprises gums, the bony socket, the outer layer of the roots of teeth, and the associated connective tissue. This disease begins with the formation of plaque. Symptoms of periodontal disease are bleeding on probing, color alterations, swelling, pain, and in advanced stages, dental mobility. Probiotics prevent plaque formation by reducing the pH of saliva and producing antioxidants that use free electrons used in plaque mineralization because, in this condition, carcinogenic bacteria are not able to form plaque. Therefore, in this way, probiotics prevent periodontal disease. Also, encouraging results of the consumption of probiotics in the cure of plaque level, gingivitis, periodontitis, and significant reduction of periodontopathogens have been reported in various studies. Today, according to new knowledge, three factors are known to cause plaque-related periodontitis. These three factors are a vulnerable host, the existence of a pathogen, and the low extent or absence of advantageous microbiota (Slots and Rams, 1991; Socransky and Haffajee, 1992; Wolff et al., 1994). An unbalance between the pathogenic and saprophytic flora of the oral cavity in a vulnerable individual may cause periodontal disease (Dye, 2012). Through scaling and root planing, and deep pocket debridement, which are considered the primary cure, it is possible to reduce invasive periopathogens significantly. Debridement treatment of periodontal disease is possible in two ways, surgical or non-surgical. Sometimes, systemic antimicrobial administration is also necessary. Although plaque removal using mechanical surface instrumentation is an effective treatment method, pocket recolonization is highly unpredictable (Morales et al., 2016; Meurman and Stamatova, 2018). Considering that the administration of these drugs gradually creates bacterial resistance, new alternatives are needed for them (Laleman and Teughels, 2015; Morales et al., 2016). Since a probiotic can modify the oral microbial flora, it is probably a purposive strategy in the clinical control of periodontitis in addition to other beneficial advantages it provides to the host (Saha et al., 2012). On the one hand, probiotics can compete with periodontal pathogens and modulate dysbiosis conditions, therewith decreasing the overall immunogenicity of the oral microbiota, and also, they can modulate immune/inflammatory pathways to decrease the destructive inflammation of periodontitis and result in immune homeostasis that can be retained by the individual for a long time (Allaker and Stephen, 2017). It has been shown that the existence of probiotics with a suitable concentration of 10^8^ CFU/mL in periodontal dressings reduces the number of periodontal pathogens, including *Actinomyces* sp., *Bacteroides* sp., *S. intermedius*, and *C. albicans* (Volozhin et al., 2004). The impact of probiotic *Lactobacilli* on inhibiting the growth of periodontopathogens in the oral cavity has also been shown (Sookkhee et al., 2001; Ishikawa et al., 2003; Kõll‐Klais et al., 2005b). There is a straight relationship between periodontal inflammation and destruction and the reduction of LAB levels (Kõll-Klais et al., 2005a). It has been reported that bacteria such as *L. fermentum* and *L. gasseri* in the oral cavity of patients with chronic periodontitis had lower levels than in healthy people (Kõll‐Klais et al., 2005b). The usage of *L. reuteri* has also shown a decrease in gum bleeding and a decrease in gum inflammation (Krasse et al., 2006; Meurman and Stamatova, 2007). In another study, *L. reuteri* was used as a supplement to conventional treatments for patients with periodontitis, and it caused a decrease in the number of *P. gingivalis, P. intermedia*, and *Aggregatibacter actinomycetemcomitans* (Vivekananda et al., 2010). In addition to reducing the count of periodontal pathogens, the activity of probiotics has resulted in a decrease in the concentration of IL-17, IL-1β, and TNF-α in the gingival crevice fluid (Teughels et al., 2013; Szkaradkiewicz et al., 2014). It has been shown in mouse models that gut-based probiotics can have a protective effect on periodontitis through immune modulation (McCabe et al., 2015; Kobayashi et al., 2017). Therefore, this characteristic of probiotics can make them an alternative to antibiotics in periodontal cure to help decrease the overall burden of antibiotic resistance (Bidault et al., 2007; Rams et al., 2014). Meta-analyses also support the consumption of probiotics in the management of periodontitis (Gruner et al., 2016; Martin-Cabezas et al., 2016). However, before recommending the routine consumption of probiotics for the cure of periodontitis and gingivitis, various aspects and points should be considered. For example, the length of the treatment and how to do it so that the pathogenic microbiota does not prevail again after it ends, and also pay attention to the possible risks that the consumption of probiotics can cause in subjects with a weak immune system.

## 7 Probiotics and halitosis

**Figure 3:**
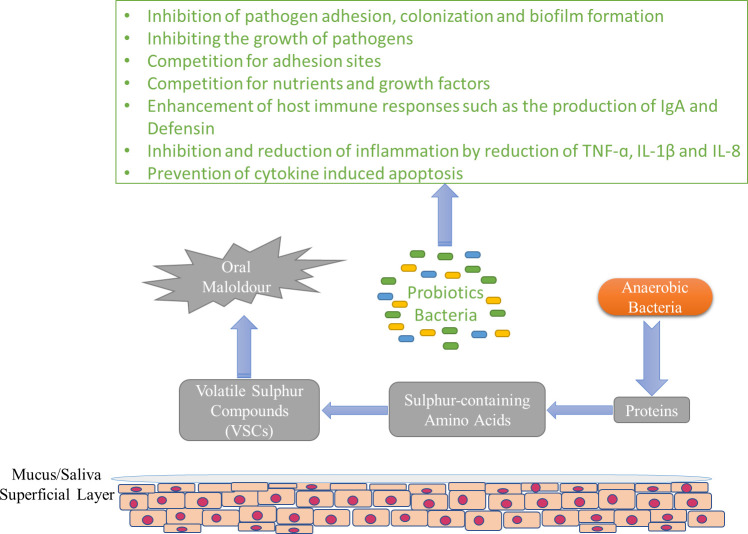
The general pattern of formation of VSCs and possible mechanisms of probiotics in the prevention of halitosis.

Halitosis, or the nasty odor that comes out of the mouth, is a disease that depends on various factors, and its origin may be oral or non-oral (Van den Broek et al., 2007; Oliveira-Neto et al., 2013). Halitosis is usually attributed to biofilm in the interdental spaces, the back of the tongue, and chronic inflammatory diseases. The incidence of this disease in different populations is estimated between 22 and 50 percent (Miyazaki, 1995; Meningaud et al., 1999; Yaegaki and Coil, 2000; Quirynen et al., 2009; Akaji et al., 2014). Sulfuric gases such as dimethyl sulfide, hydrogen sulfide, and methyl mercaptan play an important role in causing bad breath. These gases are released in the oropharynx (tongue coating, tonsillitis, gingivitis, periodontitis) through bacterial degradation of sulfur-containing amino acids (**Figure 3**). *F. nucleatum*, *Treponema denticola*, *P. intermedia*, and *P. gingivalis* can be mentioned among the diverse range of bacteria that contribute to this disease (Corcoran et al., 2004). On the other hand, the levels of bacterial species that form the dominant microbiota in the oral of healthy people are not significant in people with halitosis (Kazor et al., 2003). Current treatments seek to eliminate these pathogenic bacteria using chemical or physical antibacterial agents. Antimicrobial treatment indiscriminately reduces the population of pathogenic bacteria and bacteria that are not involved in causing halitosis but are probably effective in maintaining a normal oral microenvironment. However, the result of this cure is to reduce the bad smell temporarily, and after some time, the bacteria causing halitosis appear again (Burton et al., 2005). Probiotics that are effective in maintaining periodontal health may also be useful for eliminating bad breath by helping to keep a healthy ecology of the tongue because in oral health, access to certain areas of the tongue, such as the dorsal posterior surface to the circumvallate papillae, which acts as a shelter for the large number of gram-negative bacterial species correlated with bad breath, is more difficult (Allaker et al., 2008). Nevertheless, it is known that the tongue is a more palpable recess than the periodontal recesses in terms of species that normally colonize, suggesting the necessity of recess-specific conformity, and probiotic strains intended to colonize the periodontal recesses may not freely colonize the tongue to exert health-promoting impacts (Zaura et al., 2009; Eren et al., 2014). For the first time, Kang et al. (2006)sought a probiotic to prevent or treat halitosis using a scientific approach. In children, after gargling the rinsing solution containing *Weissella cibaria*, a significant decrease in H_2_S (48.2%) and CH_3_SH (59.4%) levels, and as a result, halitosis has been observed (Lima et al., 2005; Kang et al., 2006). A possible candidate to compete with bacterial species that increase the level of volatile sulfur compounds is *S. salivarius*, which has shown an inhibitory effect on volatile sulfur compounds (VSC). Rinsing with chlorhexidine followed by lozenges containing *S. salivarius* K12 strain decreased the respiratory levels of VSCs in most subjects and maintained their levels for at least 2 weeks. (Sullivan and Nord, 2005). This strain produces two lantibiotic bacteriocins that inhibit gram-positive strains involved in halitosis (Meurman and Stamatova, 2007). *S. salivarius* K12 seems to be able to reduce bad breath for a long time by replacing the bacteria involved in halitosis (Burton et al., 2006). In some other studies, the effect of this strain in eliminating bad breath has been reported (Horz et al., 2007; Wescombe et al., 2010; Masdea et al., 2012). In a study, it was shown that *L. salivarius* WB21 has a positive effect on the organoleptic test score and bad breath (Iwamoto et al., 2010). Chewing gum containing probiotics can also reduce the levels of non-sulfur odor-producing bacteria. In the study by Keller et al. (2012b), this was mentioned, and it was shown that the organoleptic scores were significantly decreased after the consumption of chewing gum containing *L. reuteri* compared to the placebo group. In contrast, there was no improvement in breath VSCs concentration or organoleptic scores after being treated with *L. brevis* CD2 lozenges (Marchetti et al., 2015). Also, no significant decrease in breath VSCs concentration or organoleptic scores was observed after consuming milk containing *L. casei* Shirota (Sutula et al., 2013). Anti-VSC effects by non-oral bacteria such as *S. thermophilus*, *E. faecium*, and *W. cibaria* have also been reported in laboratory studies (Lee and Baek, 2014; Jang et al., 2016; Suzuki et al., 2016). It seems that the effect of probiotics, mostly used in commercial foods, has been less investigated in past studies. Therefore, it is recommended that future researches focus on such strains.

## 8 The application of postbiotics as novel biological compounds to improve oral health

**Figure 4:**
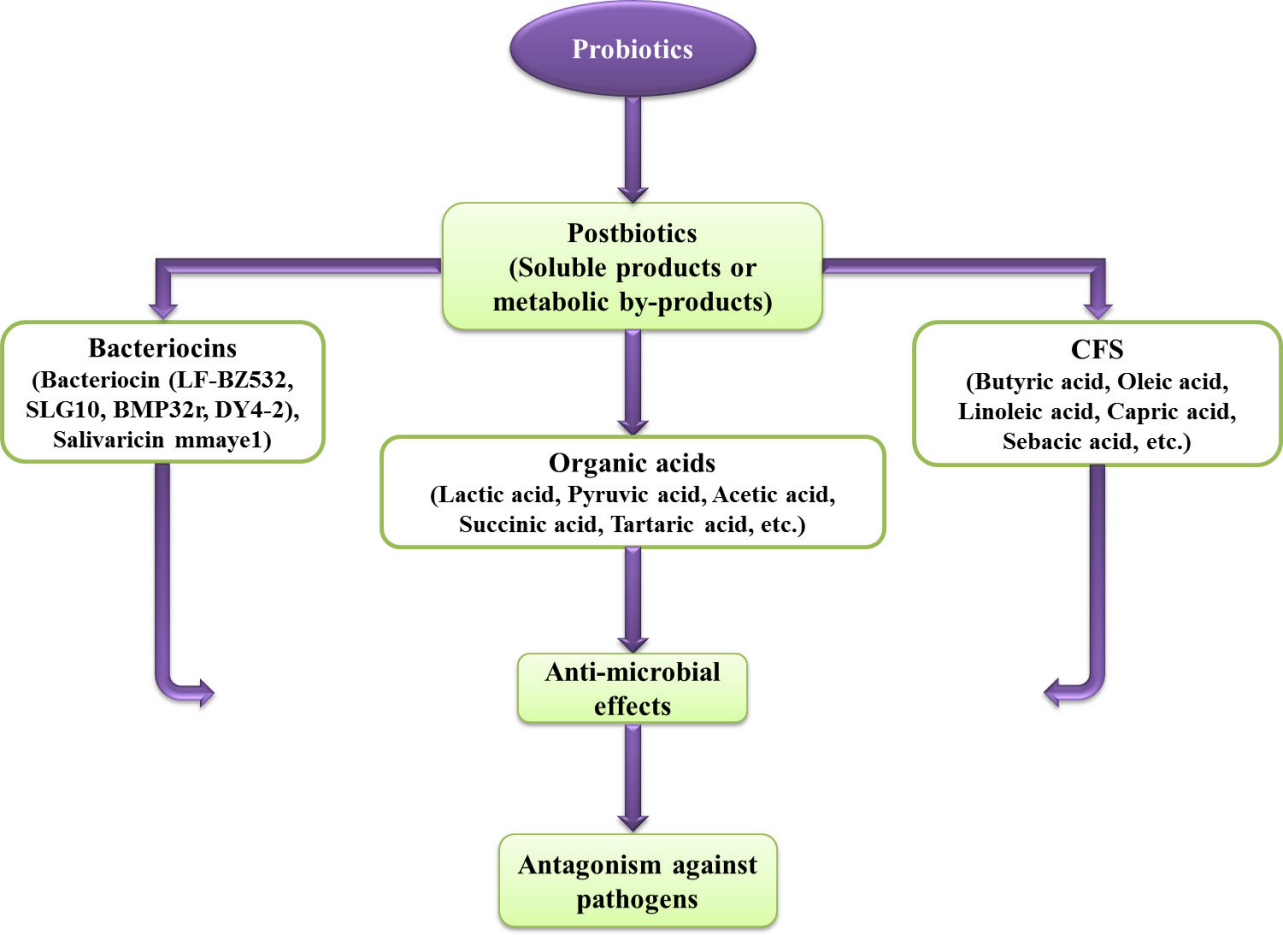
The indirect effect of probiotic bacteria through produced compounds.

During the past years, researchers have usually used different idioms such as cell-free supernatant, biogenic, abiotics, metabiotics, pseudoprobiotics, ghost probiotics, paraprobiotics, and postbiotics to introduce non-viable parts or metabolites of probiotic bacteria. In the meantime, postbiotic has been used the most (Abbasi et al., 2022). By definition, postbiotics are cell wall fragments, cytoplasmic extracts, or metabolites produced by gut-resident probiotics and fermented foods. However, because of the uncertain definition and broad bioactivity of metabolites produced by probiotics, the term postbiotic can be defined as follows: any soluble agent (products or metabolic by-products of microbial metabolisms or compounds produced by the action of LAB on culture or food ingredients) that is released by live probiotics or after their cell lysis during fermentations is secreted in food, microbiological cultures or gut. During the fermentation process, probiotics feed on prebiotics and produce a wide range of postbiotics. The functional mechanism of probiotics is related to the production of compounds like bacteriocins, organic acids, fatty acids, and hydrogen peroxide (Miles, 2007; Stamatova and Meurman, 2009; Takahashi, 2015; Żółkiewicz et al., 2020; Rad et al., 2020b; Moradi et al., 2021) (**Figure 4**). These compounds are currently produced by laboratory methods, and if they are consumed in sufficient amounts, they will have health effects (Aguilar-Toalá et al., 2018; Moradi et al., 2021). Also, due to their favorable antimicrobial effects, they are considered a promising alternative to antibiotics (Johnson et al., 2019; Rad et al., 2020a). Organic acids are acknowledged as one of the most important and impressive components of postbiotics. Lactic acid produced during bacterial fermentation effectively controls pathogenicity (Baird et al., 2006). Bacteriocins are peptides that have antimicrobial activity. These compounds resist heat and pH and can prevent the development of pathogens in the gastrointestinal. The mechanism of function of bacteriocins is in the cytoplasmic membrane of bacteria (Gálvez et al., 2007; Šušković et al., 2010). Also, fatty acids, as one of the components of postbiotics, have shown good antimicrobial effects. Long-chain fatty acids like eicosapentaenoic acid are active against gram-positive bacteria. Fatty acids exhibit various antimicrobial mechanisms against bacteria, including increasing membrane permeability, lysing bacterial cells, disrupting the structure and activity of enzymes, disrupting the electron transport chain, and inducing morphological and functional changes in susceptible components like proteins (Desbois, 2012; Yoon et al., 2018). H_2_O_2_ (Hydrogen peroxide) is another antimicrobial agent whose antimicrobial effect is said to be due to its strong oxidizing action on the bacterial cell and the destruction it causes to the structure of proteins in its cytoplasm (Osborn and Akoh, 2002). Earlier, the advantageous effects of some probiotics in improving oral health and preventing oral diseases are mentioned. It is thought that the antimicrobial properties of probiotics are because of the production of antibacterial compounds, such as hydrogen peroxide and organic acids, and antifungal compounds, such as bacteriocins and fatty acids (Twetman et al., 2009a). Also, it has been reported that *L. reuteri* produces water-soluble antimicrobial compounds like reuterin (Talarico et al., 1988) and reutericyclin (Ganzle et al., 2000), which have antagonistic activity. These substances are resistant to lipolytic and proteolytic enzymes (El-Ziney and Debevere, 1998) and maintain their function in a wide range of pH (Rodriguez et al., 2003). The strain of *S. dentisani* isolated from the mouth of people without caries also can produce bacteriocin and can be considered another useful probiotic spp. (López-López et al., 2017). Also, in a three-month clinical study, the results showed that the *S. salivarius* M18 probiotic strain, which also produces bacteriocin, can reduce the development of tooth decay in children (Di Pierro et al., 2015). It has been found that some bacterial spp., such as *S. sanguinis* (Trüper and De’Clari, 1997) and *S. uberis*, are not present in the subgingival plaque samples of people with refractory periodontitis and people with localized juvenile periodontitis. At the same time, they are present in the subgingival plaque samples of healthy individuals. It was shown that these strains prevent the growth of *A. actinomycetemcomitans* (Norskov-Lauritsen, 2006) and other periodontal pathogens (Hillman and Socransky, 1982; Hillman et al., 1985) through the production of hydrogen peroxide (Hillman and Shivers, 1988).

It has been stated that the decrease in pH level and tooth demineralization by bacteria (Kidd and Fejerskov, 2004) like *S. mutans*, *S. sobrinus*, and *Lactobacillus* spp. is because of the organic acids produced in the biofilm as metabolic byproducts produced during fermentation (Simon, 2007; Conrads et al., 2014). On the other hand, as mentioned, one of the mechanisms of probiotics to prevent the growth of pathogenic bacteria is the production of organic acids. Therefore, the question may arise whether the presence of these acids causes a further reduction in pH and an increase in tooth decay. In different studies, it was shown that salivary pH levels were significantly higher in the probiotic treatment group than in the control group. The results of this study are consistent with some other studies that have reported an increase in pH level after the use of yogurt (Shakovetz et al., 2013) and curd-containing probiotics (SrivaStava et al., 2016a). The increase in the pH level after the consumption of probiotics can be considered due to their competition with pathogenic bacteria and reducing their number. This reduction in the number of acid-producing pathogens causes the production of acid to decrease and, as a result, the pH level of saliva increases. Since tooth decay is affected by pH imbalance, these findings can be important. The issue of protection and security has been noticed in recent years due to the prevalence of probiotic food products. A probiotic strain to treat a bacterial disease must have some characteristics. For example, the probiotic bacteria should not cause disease or otherwise susceptible the individual to other disease states by disrupting the ecosystem in which it resides (Hillman et al., 2000). They should also be examined and screened for general safety, such as antibiotic resistance genes and those related to the potential invasion (Corcoran et al., 2004; Meurman and Stamatova, 2007). Considering these concerns about the consumption of live bacteria, we should consider a suitable and safe alternative that has the same biological health benefits as probiotics (Aguilar-Toalá et al., 2018). The evidence achieved from *in vitro* and *in vivo* studies indicates that postbiotics are clinically safe and have beneficial and therapeutic effects if used in appropriate amounts and duration (Patel and Denning, 2013; Dinić et al., 2017). With features such as a safe profile, long shelf life (up to 5 years), no toxic effects, standardization, and easier transportation, postbiotics can be safe and cost-effective alternatives for probiotics in the pharmaceutical and food industries (Homayouni Rad et al., 2021; Rad et al., 2021a).

## 9 Conclusion

Probiotics and their therapeutic effects in the control, prevention, and treatment of some diseases have been the subject of attention for many years. Therefore, people’s desire to use probiotic food and pharmaceutical products is increasing day by day. Due to the problems that exist in the survival of probiotics in food until the time of consumption, as well as under adverse environmental and gastrointestinal conditions, the interest in using probiotic side products and secretions, especially postbiotics, has increased. Because there is no need for survival of probiotics, and also these compounds are kept unchanged for a long time and are easily included in different food formulations, therefore, postbiotics can be a suitable alternative to replace live probiotics. One of the new topics is the use of postbiotics in maintaining and improving the health of the oral cavity. Various studies have shown that the presence of probiotics in the oral cavity has significant effects in reducing abnormalities such as tooth decay, oral cavity fungus, infection, and swelling of the gums and palate. It seems that the use of postbiotics instead of probiotics has a similar effect and can be effective in the prevention and cure of many oral and dental infectious diseases caused by the presence of pathogens such as *S. mutans*, and the results of existing studies confirm this impact. However, there are still some challenges as to whether postbiotics are as durable as probiotics. Also, in order to prove these preventive and therapeutic effects, many animal studies and human clinical trials are needed, especially in the case of postbiotics of different types and species of probiotics, to be able to prove their effectiveness finally. Since probiotics are acidogenic like cariogenic bacteria such as *S. mutans*, future studies also could examine the issue of what differences exist between the amount and type of acids produced by probiotic and pathogenic strains. The results of these studies can provide clearer evidence and reasons for whether there is a difference in the cariogenic of these acids and the strains that produce them.

## Author contributions

EM and AHR conceived the idea. EM wrote the first draft of the manuscript. HP wrote sections of the manuscript. All authors contributed to the manuscript revision, read, and approved the submitted version.

## References

[B1] Aas J. A. Griffen A. L. Dardis S. R. Lee A. M. Olsen I. Dewhirst F. E.. (2008). Bacteria of dental caries in primary and permanent teeth in children and young adults. J. Clin. Microbiol. 46 (4), 1407–1417. doi: 10.1128/JCM.01410-07 18216213 PMC2292933

[B2] Aas J. A. Paster B. J. Stokes L. N. Olsen I. Dewhirst F. E. (2005). Defining the normal bacterial flora of the oral cavity. J. Clin. Microbiol. 43 (11), 5721–5732. doi: 10.1128/JCM.43.11.5721-5732.2005 16272510 PMC1287824

[B3] Abbasi A. Rad A. H. Ghasempour Z. Sabahi S. Kafil H. S. Hasannezhad P.. (2022). The biological activities of postbiotics in gastrointestinal disorders. Crit. Rev. Food Sci. Nutr. 62 (22), 5983–6004. doi: 10.1080/10408398.2021.1895061 33715539

[B4] Abd S. T. Ali A. F. (2016). The effect of zinc oxide nanoparticles on streptococcus mutans of human saliva (*In vitro* study). J. Baghdad. Coll. Dentistry. 28 (2), 158–164.

[B5] Aguilar-Toalá J. Garcia-Varela R. Garcia H. Mata-Haro V. González-Córdova A. Vallejo-Cordoba B.. (2018). Postbiotics: An evolving term within the functional foods field. Trends Food Sci. Technol. 75, 105–114. doi: 10.1016/j.tifs.2018.03.009

[B6] Ahn J. Yang L. Paster B. J. Ganly I. Morris L. Pei Z.. (2011). Oral microbiome profiles: 16S rRNA pyrosequencing and microarray assay comparison. PloS One 6 (7), e22788. doi: 10.1371/journal.pone.0022788 21829515 PMC3146496

[B7] Ahola A. J. Yli-Knuuttila H. Suomalainen T. Poussa T. Ahlström A. Meurman J. H.. (2002). Short-term consumption of probiotic-containing cheese and its effect on dental caries risk factors. Arch. Oral. Biol. 47 (11), 799–804. doi: 10.1016/S0003-9969(02)00112-7 12446187

[B8] Akaji E. A. Folaranmi N. Ashiwaju O. (2014). Halitosis: a review of the literature on its prevalence, impact and control. Oral. Health Prev. Dent. 12 (4), 297–304. doi: 10.3290/j.ohpd.a33135 25525639

[B9] Allaker R. P. Stephen A. S. (2017). Use of probiotics and oral health. Curr. Oral. Health Rep. 4 (4), 309–318. doi: 10.1007/s40496-017-0159-6 29201598 PMC5688201

[B10] Allaker R. P. Waite R. D. Hickling J. North M. McNab R. Bosma M. P. (2008). Topographic distribution of bacteria associated with oral malodour on the tongue. Arch. Oral. Biol. 53, S8–S12. doi: 10.1016/S0003-9969(08)70003-7 18460402

[B11] Aminabadi N. Erfanparast L. Ebrahimi A. Oskouei S. (2011). Effect of chlorhexidine pretreatment on the stability of salivary lactobacilli probiotic in six-to twelve-year-old children: A randomized controlled trial. Caries. Res. 45 (2), 148–154. doi: 10.1159/000325741 21454978

[B12] Angarita-Díaz M. Forero-Escobar D. Cerón-Bastidas X. Cisneros-Hidalgo C. Dávila-Narvaez F. Bedoya-Correa C.. (2020). Effects of a functional food supplemented with probiotics on biological factors related to dental caries in children: a pilot study. Eur. Arch. Paediatric. Dentistry 21 (1), 161–169. doi: 10.1007/s40368-019-00468-y 31388942

[B13] Ashwin D. Vijayaprasad K. Taranath M. Ramagoni N. K. Nara A. Sarpangala M. (2015). Effect of probiotic containing ice-cream on salivary mutans streptococci (SMS) levels in children of 6-12 years of age: A randomized controlled double blind study with six-months follow up. J. Clin. Diagn. Res.: JCDR 9 (2), ZC06. doi: 10.7860/JCDR/2015/10942.5532 PMC4378797 25859515

[B14] Bafna H. P. Ajithkrishnan C. Kalantharakath T. Singh R. P. Kalyan P. Vathar J. B.. (2018). Effect of short-term consumption of amul probiotic yogurt containing lactobacillus acidophilus La5 and bifidobacterium lactis Bb12 on salivary streptococcus mutans count in high caries risk individuals. Int. J. Appl. Basic. Med. Res. 8 (2), 111. doi: 10.4103/ijabmr.IJABMR_447_16 29744324 PMC5932918

[B15] Baird B. Lucia L. Acuff G. Harris K. Savell J. (2006). Beef hide antimicrobial interventions as a means of reducing bacterial contamination. Meat. Sci. 73 (2), 245–248. doi: 10.1016/j.meatsci.2005.11.023 22062295

[B16] Beighton D. (2005). The complex oral microflora of high-risk individuals and groups and its role in the caries process. Community Dentistry Oral. Epidemiol. 33 (4), 248–255. doi: 10.1111/j.1600-0528.2005.00232.x 16008631

[B17] Bidault P. Chandad F. Grenier D. (2007). Risk of bacterial resistance associated with systemic antibiotic therapy in periodontology. J. Can. Dental Assoc. 73 (8). 17949540

[B18] Bik E. M. Long C. D. Armitage G. C. Loomer P. Emerson J. Mongodin E. F.. (2010). Bacterial diversity in the oral cavity of 10 healthy individuals. ISME. J. 4 (8), 962–974. doi: 10.1038/ismej.2010.30 20336157 PMC2941673

[B19] Boyar R. Thylstrup A. Holmen L. Bowden G. (1989). The microflora associated with the development of initial enamel decalcification below orthodontic bands *in vivo* in children living in a fluoridated-water area. J. Dental Res. 68 (12), 1734–1738. doi: 10.1177/00220345890680120301 2600252

[B20] Bradshaw D. Marsh P. (1998). Analysis of pH–driven disruption of oral microbial communities *in vitro* . Caries. Res. 32 (6), 456–462. doi: 10.1159/000016487 9745120

[B21] Burton J. Chilcott C. Moore C. Speiser G. Tagg J. (2006). A preliminary study of the effect of probiotic streptococcus salivarius K12 on oral malodour parameters. J. Appl. Microbiol. 100 (4), 754–764. doi: 10.1111/j.1365-2672.2006.02837.x 16553730

[B22] Burton J. Chilcott C. Tagg J. (2005). The rationale and potential for the reduction of oral malodour using streptococcus salivarius probiotics. Oral. Dis. 11, 29–31. doi: 10.1111/j.1601-0825.2005.01084.x 15752094

[B23] Burton J. P. Wescombe P. A. Macklaim J. M. Chai M. H. MacDonald K. Hale J. D.. (2013). Persistence of the oral probiotic streptococcus salivarius M18 is dose dependent and megaplasmid transfer can augment their bacteriocin production and adhesion characteristics. PloS One 8 (6), e65991. doi: 10.1371/journal.pone.0065991 23785463 PMC3681767

[B24] Cagetti M. G. Mastroberardino S. Milia E. Cocco F. Lingström P. Campus G. (2013). The use of probiotic strains in caries prevention: A systematic review. Nutrients 5 (7), 2530–2550. doi: 10.3390/nu5072530 23857225 PMC3738986

[B25] Caglar E. (2014). Effect of bifidobacterium bifidum containing yoghurt on dental plaque bacteria in children. J. Clin. Pediatr. Dentistry 38 (4), 329–332. doi: 10.17796/jcpd.38.4.p608312353256684 25571684

[B26] Caglar E. Kavaloglu S. Kuscu O. Sandalli N. Holgerson P. Twetman S. (2007). Effect of chewing gums containing xylitol or probiotic bacteria on salivary mutans streptococci and lactobacilli. Clin. Oral. Invest. 11 (4), 425–429. doi: 10.1007/s00784-007-0129-9 17574481

[B27] Çaglar E. Kavaloglu Cildir S. Ergeneli S. Sandalli N. Twetman S. (2006). Salivary mutans streptococci and lactobacilli levels after ingestion of the probiotic bacterium lactobacillus reuteri ATCC 55730 by straws or tablets. Acta Odontol. Scand. 64 (5), 314–318. doi: 10.1080/00016350600801709 16945898

[B28] Çaglar E. Kuscu O. O. Cildir S. K. Kuvvetli S. S. Sandalli N. (2008a). A probiotic lozenge administered medical device and its effect on salivary mutans streptococci and lactobacilli. Int. J. Paediatric. Dentistry 18 (1), 35–39. doi: 10.1111/j.1365-263X.2007.00866.x 18086024

[B29] Caglar E. Onder Kuscu O. Selvi Kuvvetli S. Kavaloglu Cildir S. Sandalli N. Twetman S. (2008b). Short-term effect of ice-cream containing bifidobacterium lactis bb-12 on the number of salivary mutans streptococci and lactobacilli. Acta Odontol. Scand. 66 (3), 154–158. doi: 10.1080/00016350802089467 18568474

[B30] Çaglar E. Sandalli N. Twetman S. Kavaloglu S. Ergeneli S. Selvi S. (2005). Effect of yogurt with bifidobacterium DN-173 010 on salivary mutans streptococci and lactobacilli in young adults. Acta Odontol. Scand. 63 (6), 317–320. doi: 10.1080/00016350510020070 16512103

[B31] Campus G. Cocco F. Carta G. Cagetti M. G. Simark-Mattson C. Strohmenger L.. (2014). Effect of a daily dose of lactobacillus brevis CD2 lozenges in high caries risk schoolchildren. Clin. Oral. Invest. 18 (2), 555–561. doi: 10.1007/s00784-013-0980-9 PMC3936134 23644602

[B32] Cannon M. Trent B. Vorachek A. Kramer S. Esterly R. (2013). Effectiveness of CRT at measuring the salivary level of bacteria in caries prone children with probiotic therapy. J. Clin. Pediatr. Dentistry 38 (1), 55–60. doi: 10.17796/jcpd.38.1.b481624264142082 24579284

[B33] Caufield P. Schön C. Saraithong P. Li Y. Argimón S. (2015). Oral lactobacilli and dental caries: a model for niche adaptation in humans. J. Dental Res. 94 (9_suppl), 110S–118S. doi: 10.1177/0022034515576052 PMC4547204 25758458

[B34] Cheng Y. Liu J. Ling Z. (2021). Short-chain fatty acids-producing probiotics: A novel source of psychobiotics. Crit. Rev. Food Sci. Nutr. 1-31, 7929–7959. doi: 10.1080/10408398.2021.1920884 33955288

[B35] Chhour K.-L. Nadkarni M. A. Byun R. Martin F. E. Jacques N. A. Hunter N. (2005). Molecular analysis of microbial diversity in advanced caries. J. Clin. Microbiol. 43 (2), 843–849. doi: 10.1128/JCM.43.2.843-849.2005 15695690 PMC548050

[B36] Chuang L.-C. Huang C.-S. Ou-Yang L.-W. Lin S.-Y. (2011). Probiotic lactobacillus paracasei effect on cariogenic bacterial flora. Clin. Oral. Invest. 15 (4), 471–476. doi: 10.1007/s00784-010-0423-9 PMC3133768 20502929

[B37] Cildir S. K. Sandalli N. Nazli S. Alp F. Caglar E. (2012). A novel delivery system of probiotic drop and its effect on dental caries risk factors in cleft lip/palate children. Cleft. Palate-craniofacial. J. 49 (3), 369–372. doi: 10.1597/10-035 21309653

[B38] Cogulu D. Topaloglu-Ak A. Caglar E. Sandalli N. Karagozlu C. Ersin N.. (2010). Potential effects of a multistrain probiotic-kefir on salivary streptococcus mutans and lactobacillus spp. J. Dental Sci. 5 (3), 144–149. doi: 10.1016/S1991-7902(10)60021-9

[B39] Collaborators G. O. D. Bernabe E. Marcenes W. Hernandez C. Bailey J. Abreu L.. (2020). Global, regional, and national levels and trends in burden of oral conditions from 1990 to 2017: A systematic analysis for the global burden of disease 2017 study. J. Dental Res. 99 (4), 362–373. doi: 10.1177/0022034520908533 PMC7088322 32122215

[B40] Comelli E. M. Guggenheim B. Stingele F. Neeser J. R. (2002). Selection of dairy bacterial strains as probiotics for oral health. Eur. J. Oral. Sci. 110 (3), 218–224. doi: 10.1034/j.1600-0447.2002.21216.x 12120707

[B41] Conrads G. de Soet J. J. Song L. Henne K. Sztajer H. Wagner-Döbler I.. (2014). Comparing the cariogenic species streptococcus sobrinus and s. mutans on whole genome level. J. Oral. Microbiol. 6 (1), 26189. doi: 10.3402/jom.v6.26189 25475081 PMC4256546

[B42] Corby P. Lyons-Weiler J. Bretz W. Hart T. Aas J. Boumenna T.. (2005). Microbial risk indicators of early childhood caries. J. Clin. Microbiol. 43 (11), 5753–5759. doi: 10.1128/JCM.43.11.5753-5759.2005 16272513 PMC1287835

[B43] Corcoran B. Ross R. Fitzgerald G. Stanton C. (2004). Comparative survival of probiotic lactobacilli spray-dried in the presence of prebiotic substances. J. Appl. Microbiol. 96 (5), 1024–1039. doi: 10.1111/j.1365-2672.2004.02219.x 15078519

[B44] Deepti A. Jeevarathan J. Muthu M. (2008). Effect of fluoride varnish on streptococcus mutans count in saliva of caries free children using dentocult SM strip mutans test: A randomized controlled triple blind study. Int. J. Clin. Pediatr. Dentistry. 1 (1), 1. doi: 10.5005/jp-journals-10005-1001 PMC4086538 25206081

[B45] de Matos B. M. Brighenti F. L. Do T. Beighton D. Koga-Ito C. Y. (2017). Acidogenicity of dual-species biofilms of bifidobacteria and streptococcus mutans. Clin. Oral. Invest. 21 (5), 1769–1776. doi: 10.1007/s00784-016-1958-1 27660160

[B46] Desbois,. A. P. (2012). Potential applications of antimicrobial fatty acids in medicine, agriculture and other industries. Recent Patents. Anti-infective. Drug Discovery 7 (2), 111–122. doi: 10.2174/157489112801619728 22630821

[B47] Devine D. A. Marsh P. D. Meade J. (2015). Modulation of host responses by oral commensal bacteria. J. Oral. Microbiol. 7 (1), 26941. doi: 10.3402/jom.v7.26941 25661061 PMC4320998

[B48] Dierksen K. P. Moore C. J. Inglis M. Wescombe P. A. Tagg J. R. (2007). The effect of ingestion of milk supplemented with salivaricin a-producing streptococcus salivarius on the bacteriocin-like inhibitory activity of streptococcal populations on the tongue. FEMS Microbiol. Ecol. 59 (3), 584–591. doi: 10.1111/j.1574-6941.2006.00228.x 17069620

[B49] Dinić M. Lukić J. Djokić J. Milenković M. Strahinić I. Golić N.. (2017). Lactobacillus fermentum postbiotic-induced autophagy as potential approach for treatment of acetaminophen hepatotoxicity. Front. Microbiol. 8, 594. doi: 10.3389/fmicb.2017.00594 28428777 PMC5382196

[B50] Di Pierro F. Zanvit A. Nobili P. Risso P. Fornaini C. (2015). Cariogram outcome after 90 days of oral treatment with streptococcus salivarius M18 in children at high risk for dental caries: results of a randomized, controlled study. Clin. Cosmetic. Investigational. Dentistry 7, 107. doi: 10.2147/CCIDE.S93066 PMC4598214 26491371

[B51] Doel J. J. Benjamin N. Hector M. P. Rogers M. Allaker R. P. (2005). Evaluation of bacterial nitrate reduction in the human oral cavity. Eur. J. Oral. Sci. 113 (1), 14–19. doi: 10.1111/j.1600-0722.2004.00184.x 15693824

[B52] Dye B. A. (2012). Global periodontal disease epidemiology. Periodontol. 2000. 58 (1), 10–25. doi: 10.1111/j.1600-0757.2011.00413.x 22133364

[B53] Edwardsson S. (1974). Bacteriological studies on deep areas of carious dentine. Odontol. Rev. 25 (32), 1–143. 4614155

[B54] El-Ziney M. Debevere J. (1998). The effect of reuterin on listeria monocytogenes and escherichia coli O157: H7 in milk and cottage cheese. J. Food Prot. 61 (10), 1275–1280. doi: 10.4315/0362-028X-61.10.1275 9798141

[B55] Eren A. M. Borisy G. G. Huse S. M. Mark Welch J. L. (2014). Oligotyping analysis of the human oral microbiome. Proc. Natl. Acad. Sci. 111 (28), E2875–E2884. doi: 10.1073/pnas.1409644111 24965363 PMC4104879

[B56] Fitzgerald R. J. Keyes P. H. (1960). Demonstration of the etiologic role of streptococci in experimental caries in the hamster. J. Am. Dental Assoc. 61 (1), 9–19. doi: 10.14219/jada.archive.1960.0138 13823312

[B57] Flichy Fernández A. Alegre Domingo T. Peñarrocha Oltra D. Peñarrocha Diago M. (2010). Probiotic treatment in the oral cavity: An update. Med Oral Patol Oral Cir Bucal. 15 (5), 677–80. 10.4317/medoral.15.e677 20173706

[B58] Gálvez A. Abriouel H. López R. L. Omar N. B. (2007). Bacteriocin-based strategies for food biopreservation. Int. J. Food Microbiol. 120 (1-2), 51–70. doi: 10.1016/j.ijfoodmicro.2007.06.001 17614151

[B59] Ganzle M. G. Holtzel A. Walter J. Jung G. N. Hammes W. P. (2000). Characterization of reutericyclin produced by lactobacillus reuteri LTH2584. Appl. Environ. Microbiol. 66 (10), 4325–4333. doi: 10.1128/AEM.66.10.4325-4333.2000 11010877 PMC92303

[B60] Ghasemi E. Mazaheri R. Tahmourespour A. (2017). Effect of probiotic yogurt and xylitol-containing chewing gums on salivary s mutans count. J. Clin. Pediatr. Dentistry. 41 (4), 257–263. doi: 10.17796/1053-4628-41.4.257 28650782

[B61] Ghasempour M. Sefidgar S. A. A. Moghadamnia A. A. Ghadimi R. Gharekhani S. Shirkhani L. (2014). Comparative study of kefir yogurt-drink and sodium fluoride mouth rinse on salivary mutans streptococci. J. Contemp. Dental Pract. 15 (2), 214. 10.5005/jp-journals-10024-1517 25095846

[B62] Gill S. R. Pop M. DeBoy R. T. Eckburg P. B. Turnbaugh P. J. Samuel B. S.. (2006). Metagenomic analysis of the human distal gut microbiome. Science 312 (5778), 1355–1359. doi: 10.1126/science.1124234 16741115 PMC3027896

[B63] Gizani S. Petsi G. Twetman S. Caroni C. Makou M. Papagianoulis L. (2016). Effect of the probiotic bacterium lactobacillus reuteri on white spot lesion development in orthodontic patients. Eur. J. Orthodontics. 38 (1), 85–89. doi: 10.1093/ejo/cjv015 25840585

[B64] Glavina D. Goršeta K. Škrinjarić I. Negovetić Vranić D. Mehulić K. Kožul K. (2012). Effect of LGG yoghurt on streptococcus mutans and lactobacillus spp. salivary counts in children. Collegium. Antropol. 36 (1), 129–132. 22816209

[B65] Gomar-Vercher S. Cabrera-Rubio R. Mira A. Almerich-Silla J. (2014). Relationship of children’s salivary microbiota with their caries status: a pyrosequencing study. Clin. Oral. Invest. 18 (9), 2087–2094. doi: 10.1007/s00784-014-1200-y 24532386

[B66] Gruner D. Paris S. Schwendicke F. (2016). Probiotics for managing caries and periodontitis: Systematic review and meta-analysis. J. Dentistry. 48, 16–25. doi: 10.1016/j.jdent.2016.03.002 26965080

[B67] Gupta D. Gupta R. K. (2015). Investigation of antibacterial efficacy of acacia nilotica against salivary mutans streptococci: A randomized control trial. Gen. Dentistry. 63 (1), 23–27. 25574715

[B68] Han N. Jia L. Su Y. Du J. Guo L. Luo Z.. (2019). Lactobacillus reuteri extracts promoted wound healing *via* PI3K/AKT/β-catenin/TGFβ1 pathway. Stem Cell Res. Ther. 10 (1), 1–11. doi: 10.1186/s13287-019-1324-8 31391121 PMC6686392

[B69] Hardie J. Thomson P. South R. Marsh P. Bowden G. McKee A.. (1977). A longitudinal epidemiological study on dental plaque and the development of dental caries–interim results after two years. J. Dental Res. 56 (3_suppl), 90–98. doi: 10.1177/00220345770560032401 273035

[B70] Hasslöf P. Stecksén-Blicks C. (2020). Probiotic bacteria and dental caries. Impact. Nutr. Diet. Oral. Health 28, 99–107. doi: 10.1159/000455377 31940624

[B71] Hasslöf P. West C. Videhult F. K. Brandelius C. Stecksén-Blicks C. (2013). Early intervention with probiotic lactobacillus paracasei F19 has no long-term effect on caries experience. Caries. Res. 47 (6), 559–565. doi: 10.1159/000350524 23838478

[B72] Hatakka K. Ahola A. J. Yli-Knuuttila H. Richardson M. Poussa T. Meurman J. H.. (2007). Probiotics reduce the prevalence of oral candida in the elderly–a randomized controlled trial. J. Dental Res. 86 (2), 125–130. doi: 10.1177/154405910708600204 17251510

[B73] Hatakka K. Savilahti E. Pönkä A. Meurman J. H. Poussa T. Näse L.. (2001). Effect of long term consumption of probiotic milk on infections in children attending day care centres: Double blind, randomised trial. Bmj 322 (7298), 1327. doi: 10.1136/bmj.322.7298.1327 11387176 PMC32161

[B74] Haukioja A. Söderling E. Tenovuo J. (2008). Acid production from sugars and sugar alcohols by probiotic lactobacilli and bifidobacteria *in vitro* . Caries. Res. 42 (6), 449–453. doi: 10.1159/000163020 18931494

[B75] Haukioja A. Yli-Knuuttila H. Loimaranta V. Kari K. Ouwehand A. Meurman J. H.. (2006). Oral adhesion and survival of probiotic and other lactobacilli and bifidobacteria *in vitro* . Oral. Microbiol. Immunol. 21 (5), 326–332. doi: 10.1111/j.1399-302X.2006.00299.x 16922933

[B76] Hedberg M. Hasslöf P. Sjöström I. Twetman S. Stecksén-Blicks C. (2008). Sugar fermentation in probiotic bacteria–an *in vitro* study. Oral. Microbiol. Immunol. 23 (6), 482–485. doi: 10.1111/j.1399-302X.2008.00457.x 18954354

[B77] Hillman J. Brooks T. Michalek S. Harmon C. Snoep J. van der Weijden C. (2000). Construction and characterization of an effector strain of streptococcus mutans for replacement therapy of dental caries. Infect. Immun. 68 (2), 543–549. doi: 10.1128/IAI.68.2.543-549.2000 10639415 PMC97174

[B78] Hillman E. T. Lu H. Yao T. Nakatsu C. H. (2017). Microbial ecology along the gastrointestinal tract. Microbes Environments. 32 (4), 300–313. doi: 10.1264/jsme2.ME17017 29129876 PMC5745014

[B79] Hillman J. Shivers M. (1988). Interaction between wild-type, mutant and revertant forms of the bacterium streptococcus sanguis and the bacterium actinobacillus actinomycetemcomitans *in vitro* and in the gnotobiotic rat. Arch. Oral. Biol. 33 (6), 395–401. doi: 10.1016/0003-9969(88)90196-3 3228385

[B80] Hillman J. Socransky S. (1982). Bacterial interference in the oral ecology of actinobacillus actinomycetemcomitans and its relationship to human periodontosis. Arch. Oral. Biol. 27 (1), 75–77. doi: 10.1016/0003-9969(82)90180-7 6951532

[B81] Hillman J. Socransky S. Shivers M. (1985). The relationships between streptococcal species and periodontopathic bacteria in human dental plaque. Arch. Oral. Biol. 30 (11-12), 791–795. doi: 10.1016/0003-9969(85)90133-5 3868968

[B82] Homayouni Rad A. Aghebati Maleki L. Samadi Kafil H. Abbasi A. (2021). Postbiotics: A novel strategy in food allergy treatment. Crit. Rev. Food Sci. Nutr. 61 (3), 492–499. doi: 10.1080/10408398.2020.1738333 32160762

[B83] Homayouni-Rad A. Fathi-Zavoshti H. Douroud N. Shahbazi N. Abbasi A. (2020a). Evaluating the role of postbiotics as a new generation of probiotics in health and diseases. J. Ardabil. Univ. Med. Sci. 19 (4), 381–399. doi: 10.29252/jarums.19.4.381

[B84] Homayouni Rad A. Samadi Kafil H. Fathi Zavoshti H. Shahbazi N. Abbasi A. (2020b). Therapeutically effects of functional postbiotic foods. Clin. Excellence. 10 (2), 33–52. doi: 10.15171/hpp.2020.02

[B85] Horz H. P. Meinelt A. Houben B. Conrads G. (2007). Distribution and persistence of probiotic streptococcus salivarius K12 in the human oral cavity as determined by real-time quantitative polymerase chain reaction. Oral. Microbiol. Immunol. 22 (2), 126–130. doi: 10.1111/j.1399-302X.2007.00334.x 17311636

[B86] Huang X. Schulte R. M. Burne R. A. Nascimento M. M. (2015). Characterization of the arginolytic microflora provides insights into pH homeostasis in human oral biofilms. Caries. Res. 49 (2), 165–176. doi: 10.1159/000365296 25634570 PMC4313619

[B87] Hughes C. V. Dahlan M. Papadopolou E. Loo C. Y. Pradhan N. S. Lu S. C.. (2012). Aciduric microbiota and mutans streptococci in severe and recurrent severe early childhood caries. Pediatr. Dentistry 34 (2), 16E–23E. PMC3353719 22583872

[B88] Iheozor-Ejiofor Z. Worthington H. V. Walsh T. O'Malley L. Clarkson J. E. Macey R.. (2015). Water fluoridation for the prevention of dental caries. Cochrane Database Syst. Rev. 6). doi: 10.1002/14651858.CD010856.pub2 PMC6953324 26092033

[B89] Ishikawa H. Aiba Y. Nakanishi M. Oh-hashi Y. Koga Y. (2003). Suppression of periodontal pathogenic bacteria in the saliva of humans by the administration of lactobacillus salivarius TI 2711. Nihon. Shishubyo. Gakkai. Kaishi. (J. Japanese. Soc. Periodontol.) 45 (1), 105–112. doi: 10.2329/perio.45.105

[B90] Iwamoto T. Suzuki N. Tanabe K. Takeshita T. Hirofuji T. (2010). Effects of probiotic lactobacillus salivarius WB21 on halitosis and oral health: an open-label pilot trial. Oral. Surge. Oral. Med. Oral. Pathol. Oral. Radiol. Endodontol. 110 (2), 201–208. doi: 10.1016/j.tripleo.2010.03.032 20659698

[B91] James K. MacDonald K. Chanyi R. Cadieux P. Burton J. (2016). Inhibition of candida albicans biofilm formation and modulation of gene expression by probiotic cells and supernatant. J. Med. Microbiol. 65 (4), 328–336. doi: 10.1099/jmm.0.000226 26847045

[B92] Jang H.-J. Kang M.-S. Yi S.-H. Hong J.-Y. Hong S.-P. (2016). Comparative study on the characteristics of weissella cibaria CMU and probiotic strains for oral care. Molecules 21 (12), 1752. doi: 10.3390/molecules21121752 27999400 PMC6274271

[B93] Jäsberg H. Söderling E. Endo A. Beighton D. Haukioja A. (2016). Bifidobacteria inhibit the growth of porphyromonas gingivalis but not of streptococcus mutans in an *in vitro* biofilm model. Eur. J. Oral. Sci. 124 (3), 251–258. doi: 10.1111/eos.12266 27061393

[B94] Javid A. Z. Amerian E. Basir L. Ekrami A. Haghighizadeh M. H. Maghsoumi-Norouzabad L. (2020). Effects of the consumption of probiotic yogurt containing bifidobacterium lactis Bb12 on the levels of streptococcus mutans and lactobacilli in saliva of students with initial stages of dental caries: A double-blind randomized controlled trial. Caries. Res. 54 (1), 68–74. doi: 10.1159/000504164 31821997

[B95] Jayaram P. Chatterjee A. Raghunathan V. (2016). Probiotics in the treatment of periodontal disease: a systematic review. J. Indian Soc. Periodontol. 20 (5), 488. doi: 10.4103/0972-124X.207053 29242683 PMC5676329

[B96] Jiang Q. Stamatova I. Kainulainen V. Korpela R. Meurman J. H. (2016). Interactions between lactobacillus rhamnosus GG and oral micro-organisms in an *in vitro* biofilm model. BMC Microbiol. 16 (1), 1–11. doi: 10.1186/s12866-016-0759-7 27405227 PMC4942979

[B97] Jiang Q. Stamatova I. Kari K. Meurman J. (2015). Inhibitory activity *in vitro* of probiotic lactobacilli against oral candida under different fermentation conditions. Beneficial. Microbes 6 (3), 361–368. doi: 10.3920/BM2014.0054 25380800

[B98] Jindal G. Pandey R. Agarwal J. Singh M. (2011). A comparative evaluation of probiotics on salivary mutans streptococci counts in Indian children. Eur. Arch. Paediatric. Dentistry. 12 (4), 211–215. doi: 10.1007/BF03262809 21806906

[B99] Johansson I. Witkowska E. Kaveh B. Lif Holgerson P. Tanner A. (2016). The microbiome in populations with a low and high prevalence of caries. J. Dental Res. 95 (1), 80–86. doi: 10.1177/0022034515609554 PMC4700664 26442950

[B100] Johnson C. N. Kogut M. H. Genovese K. He H. Kazemi S. Arsenault R. J. (2019). Administration of a postbiotic causes immunomodulatory responses in broiler gut and reduces disease pathogenesis following challenge. Microorganisms 7 (8), 268. doi: 10.3390/microorganisms7080268 31426502 PMC6723925

[B101] Jose J. E. Padmanabhan S. Chitharanjan A. B. (2013). Systemic consumption of probiotic curd and use of probiotic toothpaste to reduce streptococcus mutans in plaque around orthodontic brackets. Am. J. Orthodontics. Dentofacial. Orthopedics. 144 (1), 67–72. doi: 10.1016/j.ajodo.2013.02.023 23810047

[B102] Juneja A. Kakade A. (2012). Evaluating the effect of probiotic containing milk on salivary mutans streptococci levels. J. Clin. Pediatr. Dentistry. 37 (1), 9–14. doi: 10.17796/jcpd.37.1.tq91178m7w876644 23342560

[B103] Kang M. S. Kim B. G. Chung J. Lee H. C. Oh J. S. (2006). Inhibitory effect of weissella cibaria isolates on the production of volatile sulphur compounds. J. Clin. periodontol. 33 (3), 226–232. doi: 10.1111/j.1600-051X.2006.00893.x 16489950

[B104] Karpiński T. M. Szkaradkiewicz A. K. (2013). Microbiology of dental caries. J. Biol. Earth Sci. 3 (1), M21–M24.

[B105] Kaur K. Nekkanti S. Madiyal M. Choudhary P. (2018). Effect of chewing gums containing probiotics and xylitol on oral health in children: A randomized controlled trial. J. Int. Oral. Health 10 (5), 237. doi: 10.4103/jioh.jioh_170_18

[B106] Kaye E. K. (2017). Daily intake of probiotic lactobacilli may reduce caries risk in young children. J. Evidence. Based. Dental Pract. 17 (3), 284–286. doi: 10.1016/j.jebdp.2017.07.005 28865830

[B107] Kazor C. Mitchell P. Lee A. Stokes L. Loesche W. Dewhirst F.. (2003). Diversity of bacterial populations on the tongue dorsa of patients with halitosis and healthy patients. J. Clin. Microbiol. 41 (2), 558–563. doi: 10.1128/JCM.41.2.558-563.2003 12574246 PMC149706

[B108] Keller M. K. Bardow A. Jensdottir T. Lykkeaa J. Twetman S. (2012b). Effect of chewing gums containing the probiotic bacterium lactobacillus reuteri on oral malodour. Acta Odontol. Scand. 70 (3), 246–250. doi: 10.3109/00016357.2011.640281 22182258

[B109] Keller M. Hasslöf P. Dahlén G. Stecksén-Blicks C. Twetman S. (2012a). Probiotic supplements (Lactobacillus reuteri DSM 17938 and ATCC PTA 5289) do not affect regrowth of mutans streptococci after full-mouth disinfection with chlorhexidine: a randomized controlled multicenter trial. Caries. Res. 46 (2), 140–146. doi: 10.1159/000337098 22472585

[B110] Keller M. Nøhr Larsen I. Karlsson I. Twetman S. (2014). Effect of tablets containing probiotic bacteria (Lactobacillus reuteri) on early caries lesions in adolescents: a pilot study. Beneficial. Microbes 5 (4), 403–407. doi: 10.3920/BM2013.0089 24889893

[B111] Keller M. K. Twetman S. (2012). Acid production in dental plaque after exposure to probiotic bacteria. BMC Oral. Health 12 (1), 1–6. doi: 10.1186/1472-6831-12-44 23092239 PMC3504569

[B112] Khushbu Y. Satyam P. (2016). Dental caries: A review. Asian J. Biomed. Pharm. Sci. 6 (53), 1–7.

[B113] Kidd E. Fejerskov O. (2004). What constitutes dental caries? histopathology of carious enamel and dentin related to the action of cariogenic biofilms. J. Dental Res. 83 (1_suppl), 35–38. doi: 10.1177/154405910408301s07 15286119

[B114] Klock B. Krasse B. (1979). A comparison between different methods for prediction of caries activity. Eur. J. Oral. Sci. 87 (2), 129–139. doi: 10.1111/j.1600-0722.1979.tb00664.x 292160

[B115] Kobayashi R. Kobayashi T. Sakai F. Hosoya T. Yamamoto M. Kurita-Ochiai T. (2017). Oral administration of lactobacillus gasseri SBT2055 is effective in preventing porphyromonas gingivalis-accelerated periodontal disease. Sci. Rep. 7 (1), 1–10. doi: 10.1038/s41598-017-00623-9 28373699 PMC5428773

[B116] Kolenbrander P. E. (2000). Oral microbial communities: biofilms, interactions, and genetic systems. Annu. Rev. Microbiol. 54, 413. doi: 10.1146/annurev.micro.54.1.413 11018133

[B117] Kolenbrander P. Inouye Y. Holdeman L. (1983). New actinomyces and streptococcus coaggregation groups among human oral isolates from the same site. Infect. Immun. 41 (2), 501–506. doi: 10.1128/iai.41.2.501-506.1983 6409807 PMC264669

[B118] Kõll-Klais P. Mändar R. Leibur E. Marcotte H. Hammarström L. Mikelsaar M. (2005b). Oral lactobacilli in chronic periodontitis and periodontal health: species composition and antimicrobial activity. Oral. Microbiol. Immunol. 20 (6), 354–361. doi: 10.1111/j.1399-302X.2005.00239.x 16238595

[B119] Kõll-Klais P. Mändar R. Leibur E. Mikelsaar M. (2005a). Oral microbial ecology in chronic periodontitis and periodontal health. Microbial. Ecol. Health Dis. 17 (3), 146–155. doi: 10.1080/08910600500442891 16238595

[B120] Kraft-Bodi E. Jørgensen M. Keller M. Kragelund C. Twetman S. (2015). Effect of probiotic bacteria on oral candida in frail elderly. J. Dental Res. 94 (9_suppl), 181S–186S. doi: 10.1177/0022034515595950 26202995

[B121] Krasse P. Carlsson B. Dahl C. Paulsson A. Nilsson A. Sinkiewicz G. (2006). Decreased gum bleeding and reduced gingivitis by the probiotic lactobacillus reuteri. Swedish. Dental J. 30 (2), 55–60. 16878680

[B122] Krzyściak W. Kościelniak D. Papież M. Vyhouskaya P. Zagórska-Świeży K. Kołodziej I.. (2017). Effect of a lactobacillus salivarius probiotic on a double-species streptococcus mutans and candida albicans caries biofilm. Nutrients 9 (11), 1242. doi: 10.3390/nu9111242 29135948 PMC5707714

[B123] Laleman I. Detailleur V. Slot D. E. Slomka V. Quirynen M. Teughels W. (2014). Probiotics reduce mutans streptococci counts in humans: a systematic review and meta-analysis. Clin. Oral. Invest. 18 (6), 1539–1552. doi: 10.1007/s00784-014-1228-z 24663813

[B124] Laleman I. Teughels W. (2015). Probiotics in the dental practice: a review. Quintessence. Int. 46 (3), 255–264. 25485319 10.3290/j.qi.a33182

[B125] Laleman I. Yilmaz E. Ozcelik O. Haytac C. Pauwels M. Herrero E. R.. (2015). The effect of a streptococci containing probiotic in periodontal therapy: a randomized controlled trial. J. Clin. periodontol. 42 (11), 1032–1041. doi: 10.1111/jcpe.12464 26427036

[B126] Lee S.-H. Baek D.-H. (2014). Effects of streptococcus thermophilus on volatile sulfur compounds produced by porphyromonas gingivalis. Arch. Oral. Biol. 59 (11), 1205–1210. doi: 10.1016/j.archoralbio.2014.07.006 25105253

[B127] Lee S.-H. Kim Y.-J. (2014). A comparative study of the effect of probiotics on cariogenic biofilm model for preventing dental caries. Arch. Microbiol. 196 (8), 601–609. doi: 10.1007/s00203-014-0998-7 24919536

[B128] Lee D. K. Park S. Y. An H. M. Kim J. R. Kim M. J. Lee S. W.. (2011). Antimicrobial activity of bifidobacterium spp. isolated from healthy adult koreans against cariogenic microflora. Arch. Oral. Biol. 56 (10), 1047–1054. doi: 10.1016/j.archoralbio.2011.03.002 21439550

[B129] Lima L. M. Motisuki C. D Spolidorio M. P. Santos-Pinto L. (2005). *In vitro* evaluation of probiotics microorganisms adhesion to an artificial caries model. Eur. J. Clin. Nutr. 59 (7), 884–886. doi: 10.1038/sj.ejcn.1602158 15915155

[B130] Liu B. Faller L. L. Klitgord N. Mazumdar V. Ghodsi M. Sommer D. D.. (2012). Deep sequencing of the oral microbiome reveals signatures of periodontal disease. PloS One 7 (6), e37919. doi: 10.1371/journal.pone.0037919 22675498 PMC3366996

[B131] Loesche W. J. (1986). Role of streptococcus mutans in human dental decay. Microbiol. Rev. 50 (4), 353–380. doi: 10.1128/mr.50.4.353-380.1986 3540569 PMC373078

[B132] López-López A. Camelo-Castillo A. Ferrer M. D. Simon-Soro Á. Mira A. (2017). Health-associated niche inhabitants as oral probiotics: the case of streptococcus dentisani. Front. Microbiol. 8, 379. doi: 10.3389/fmicb.2017.00379 28344574 PMC5344910

[B133] Mahantesha T. Reddy K. P. Kumar N. P. Nara A. Ashwin D. Buddiga V. (2015). Comparative study of probiotic ice cream and probiotic drink on salivary streptococcus mutans levels in 6-12 years age group children. J. Int. Oral. Health.: JIOH. 7 (9), 47. PMC4589718 26435616

[B134] Maltz M. de Oliveira E. F. Fontanella V. Bianchi R. (2002). A clinical, microbiologic, and radiographic study of deep caries lesions after incomplete caries removal. Quintessence. Int. 33 (2), 151–159. 11890029

[B135] Marchetti E. Tecco S. Santonico M. Vernile C. Ciciarelli D. Tarantino E.. (2015). Multi-sensor approach for the monitoring of halitosis treatment *via* lactobacillus brevis (CD2)–containing lozenges–a randomized, double-blind placebo-controlled clinical trial. Sensors 15 (8), 19583–19596. doi: 10.3390/s150819583 26266414 PMC4570386

[B136] Martin-Cabezas R. Davideau J. L. Tenenbaum H. Huck O. (2016). Clinical efficacy of probiotics as an adjunctive therapy to non-surgical periodontal treatment of chronic periodontitis: a systematic review and meta-analysis. J. Clin. periodontol. 43 (6), 520–530. doi: 10.1111/jcpe.12545 26970230

[B137] Marttinen A. Haukioja A. Karjalainen S. Nylund L. Satokari R. Öhman C.. (2012). Short-term consumption of probiotic lactobacilli has no effect on acid production of supragingival plaque. Clin. Oral. Invest. 16 (3), 797–803. doi: 10.1007/s00784-011-0584-1 21732090

[B138] Masdea L. Kulik E. Hauser-Gerspach I. Ramseier A. Filippi A. Waltimo T. (2012). Antimicrobial activity of streptococcus salivarius K12 on bacteria involved in oral malodour. Arch. Oral. Biol. 57 (8), 1041–1047. doi: 10.1016/j.archoralbio.2012.02.011 22405584

[B139] Mathur S. Singh R. (2005). Antibiotic resistance in food lactic acid bacteria–a review. Int. J. Food Microbiol. 105 (3), 281–295. doi: 10.1016/j.ijfoodmicro.2005.03.008 16289406

[B140] Matsubara V. H. Wang Y. Bandara H. Mayer M. P. A. Samaranayake L. P. (2016). Probiotic lactobacilli inhibit early stages of candida albicans biofilm development by reducing their growth, cell adhesion, and filamentation. Appl. Microbiol. Biotechnol. 100 (14), 6415–6426. doi: 10.1007/s00253-016-7527-3 27087525

[B141] McCabe L. Britton R. A. Parameswaran N. (2015). Prebiotic and probiotic regulation of bone health: role of the intestine and its microbiome. Curr. Osteoporosis. Rep. 13 (6), 363–371. doi: 10.1007/s11914-015-0292-x PMC4623939 26419466

[B142] Mendonça F. H. B. P. Santos S. Faria I. Gonçalves e Silva C. R. Jorge A. O. C. Leão M. V. P. (2012). Effects of probiotic bacteria on candida presence and IgA anti-candida in the oral cavity of elderly. Braz. Dental J. 23, 534–538. doi: 10.1590/S0103-64402012000500011 23306230

[B143] Meningaud J. Bado F. Favre E. Bertrand J. C. Guilbert F. (1999). Halitosis in 1999. Rev. Stomatol. Chirurgie. Maxillo-faciale. 100 (5), 240–244. 10604216

[B144] Meurman J. H. (2005). Probiotics: do they have a role in oral medicine and dentistry? Eur. J. Oral. Sci. 113 (3), 188–196. doi: 10.1111/j.1600-0722.2005.00191.x 15953242

[B145] Meurman J. Antila H. Korhonen A. Salminen S. (1995). Effect of lactobacillus rhamnosus strain GG (ATCC 53103) on the growth of streptococcus sobrinus *in vitro* . Eur. J. Oral. Sci. 103 (4), 253–258. doi: 10.1111/j.1600-0722.1995.tb00169.x 7552958

[B146] Meurman J. H. Stamatova I. (2007). Probiotics: contributions to oral health. Oral. Dis. 13 (5), 443–451. doi: 10.1111/j.1601-0825.2007.01386.x 17714346

[B147] Meurman J. H. Stamatova I. V. (2018). Probiotics: evidence of oral health implications. Folia Med. 60 (1), 21–29. doi: 10.1515/folmed-2017-0080 29668457

[B148] Mikx F. van der Hoeven J. (1975). Symbiosis of streptococcus mutans and veillonella alcalescens in mixed continuous cultures. Arch. Oral. Biol. 20 (7), 407–410. doi: 10.1016/0003-9969(75)90224-1 1096856

[B149] Miles L. (2007). Are probiotics beneficial for health? Nutr. Bull. 32 (1), 2–5. doi: 10.1111/j.1467-3010.2007.00611.x

[B150] Mishra R. Tandon S. Rathore M. Banerjee M. (2016). Antimicrobial efficacy of probiotic and herbal oral rinses against candida albicans in children: a randomized clinical trial. Int. J. Clin. Pediatr. Dentistry. 9 (1), 25. doi: 10.5005/jp-journals-10005-1328 PMC4890058 27274151

[B151] Miyazaki H. (1995). Oral malodor in the general population of Japan. Bad. Breath.: Res. Perspect., 119–136.

[B152] Moradi M. Molaei R. Guimarães J. T. (2021). A review on preparation and chemical analysis of postbiotics from lactic acid bacteria. Enzyme Microbial. Technol. 143, 109722. doi: 10.1016/j.enzmictec.2020.109722 33375981

[B153] Morales A. Carvajal P. Silva N. Hernandez M. Godoy C. Rodriguez G.. (2016). Clinical effects of lactobacillus rhamnosus in non-surgical treatment of chronic periodontitis: a randomized placebo-controlled trial with 1-year follow-up. J. periodontol. 87 (8), 944–952. doi: 10.1902/jop.2016.150665 26944407

[B154] Mortazavi S. Akhlaghi N. (2012). Salivary streptococcus mutans and lactobacilli levels following probiotic cheese consumption in adults: A double blind randomized clinical trial. J. Res. Med. Sci. 17 (1), 57. 23248658 PMC3523439

[B155] Munson M. Banerjee A. Watson T. Wade W. (2004). Molecular analysis of the microflora associated with dental caries. J. Clin. Microbiol. 42 (7), 3023–3029. doi: 10.1128/JCM.42.7.3023-3029.2004 15243054 PMC446285

[B156] Nadelman P. Magno M. B. Masterson D. da Cruz A. G. Maia L. C. (2018). Are dairy products containing probiotics beneficial for oral health? a systematic review and meta-analysis. Clin. Oral. Invest. 22 (8), 2763–2785. doi: 10.1007/s00784-018-2682-9 30298454

[B157] Nadkarni M. A. Caldon C. E. Chhour K.-L. Fisher I. P. Martin F. E. Jacques N. A.. (2004). Carious dentine provides a habitat for a complex array of novel prevotella-like bacteria. J. Clin. Microbiol. 42 (11), 5238–5244. doi: 10.1128/JCM.42.11.5238-5244.2004 15528720 PMC525250

[B158] Nagarajappa R. Daryani H. Sharda A. J. Asawa K. Batra M. Sanadhya S.. (2015). Effect of chocobar ice cream containing bifidobacterium on salivary streptococcus mutans and lactobacilli: A randomised controlled trial. Oral. Health Prev. Dent. 13 (3), 213–218. doi: 10.3290/j.ohpd.a32673 25197733

[B159] Nikawa H. Makihira S. Fukushima H. Nishimura H. Ozaki Y. Ishida K.. (2004). Lactobacillus reuteri in bovine milk fermented decreases the oral carriage of mutans streptococci. Int. J. Food Microbiol. 95 (2), 219–223. doi: 10.1016/j.ijfoodmicro.2004.03.006 15282133

[B160] Nishihara T. Suzuki N. Yoneda M. Hirofuji T. (2014). Effects of lactobacillus salivarius-containing tablets on caries risk factors: a randomized open-label clinical trial. BMC Oral. Health 14 (1), 1–7. doi: 10.1186/1472-6831-14-110 25178882 PMC4236677

[B161] Noorda W. Purdell-Lewis D. Van Montfort A. Weerkamp A. (1988). Monobacterial and mixed bacterial plaques of streptococcus mutans and veillonella alcalescens in an artificial mouth: development, metabolism, and effect on human dental enamel. Caries. Res. 22 (6), 342–347. doi: 10.1159/000261134 3214847

[B162] Norskov-Lauritsen N. (2006). Reclassification of actinobacillus actinomycetemcomitans, haemophilus aphrophilus, haemophilus paraphrophilus and haemophilus segnis as aggregatibacter actinomycetemcomitans gen. nov., comb. nov., aggregatibacter aphraphilus comb. nov. and aggregatibacter segnis comb. nov., and emended description of aggregatibacter aphrophilus to include V factor-dependent and V factor-independent isolates. Int. J. Syst. Evol. Microbiol. 56, 2135–2146. doi: 10.1099/ijs.0.64207-0 16957111

[B163] Nozari A. Motamedifar M. Seifi N. Hatamizargaran Z. Ranjbar M. A. (2015). The effect of Iranian customary used probiotic yogurt on the children’s salivary cariogenic microflora. J. Dentistry. 16 (2), 81. PMC4445856 26046102

[B164] Oliveira-Neto J. M. Sato S. Pedrazzi V. (2013). How to deal with morning bad breath: A randomized, crossover clinical trial. J. Indian Soc. Periodontol. 17 (6), 757. doi: 10.4103/0972-124X.124497 24554886 PMC3917206

[B165] Osborn H. Akoh C. (2002). Structured lipids-novel fats with medical, nutraceutical, and food applications. Compr. Rev. Food Sci. Food Saf. 1 (3), 110–120. doi: 10.1111/j.1541-4337.2002.tb00010.x 33451231

[B166] Pahumunto N. Piwat S. Chankanka O. Akkarachaneeyakorn N. Rangsitsathian K. Teanpaisan R. (2018). Reducing mutans streptococci and caries development by lactobacillus paracasei SD1 in preschool children: a randomized placebo-controlled trial. Acta Odontol. Scand. 76 (5), 331–337. doi: 10.1080/00016357.2018.1453083 29566582

[B167] Pahumunto N. Sophatha B. Piwat S. Teanpaisan R. (2019). Increasing salivary IgA and reducing streptococcus mutans by probiotic lactobacillus paracasei SD1: a double-blind, randomized, controlled study. J. Dental Sci. 14 (2), 178–184. doi: 10.1016/j.jds.2019.01.008 PMC6562187 31210892

[B168] Palmer C. Kent R. Jr. Loo C. Hughes C. Stutius E. Pradhan N.. (2010). Diet and caries-associated bacteria in severe early childhood caries. J. Dental Res. 89 (11), 1224–1229. doi: 10.1177/0022034510376543 PMC2954266 20858780

[B169] Paster B. J. Olsen I. Aas J. A. Dewhirst F. E. (2006). The breadth of bacterial diversity in the human periodontal pocket and other oral sites. Periodontol. 2000. 42 (1), 80–87. doi: 10.1111/j.1600-0757.2006.00174.x 16930307

[B170] Patel R. M. Denning P. W. (2013). Therapeutic use of prebiotics, probiotics, and postbiotics to prevent necrotizing enterocolitis: what is the current evidence? Clinics Perinatol. 40 (1), 11–25. doi: 10.1016/j.clp.2012.12.002 PMC3575601 23415261

[B171] Petersen P. E. (2003). The world oral health report 2003: continuous improvement of oral health in the 21st century–the approach of the WHO global oral health programme. Community Dentistry. Oral. Epidemiol. 31, 3–24. doi: 10.1046/j.2003.com122.x 15015736

[B172] Petersson L. G. Magnusson K. Hakestam U. Baigi A. Twetman S. (2011). Reversal of primary root caries lesions after daily intake of milk supplemented with fluoride and probiotic lactobacilli in older adults. Acta Odontol. Scand. 69 (6), 321–327. doi: 10.3109/00016357.2011.568962 21563871

[B173] Pinto G. Cenci M. Azevedo M. Epifanio M. Jones M. (2014). Effect of yogurt containing bifidobacteriumanimalis subsp. lactis DN-173010 probiotic on dental plaque and saliva in orthodontic patients. Caries. Res. 48 (1), 63–68. doi: 10.1159/000353467 24217196

[B174] Pitts N. (2004). Are we ready to move from operative to non-operative/preventive treatment of dental caries in clinical practice? Caries. Res. 38 (3), 294–304. doi: 10.1159/000077769 15153703

[B175] Piwat S. Pahumunto N. Srisommai P. Mapaisansin C. Teanpaisan R. (2019). Effect of probiotic delivery vehicles for probiotic lactobacillus rhamnosus SD11 in caries prevention: a clinical study. J. Food Process. Preserv. 43 (10), e14147. doi: 10.1111/jfpp.14147

[B176] Piwat S. Sophatha B. Teanpaisan R. (2015). An assessment of adhesion, aggregation and surface charges of l actobacillus strains derived from the human oral cavity. Lett. Appl. Microbiol. 61 (1), 98–105. doi: 10.1111/lam.12434 25913304

[B177] Quirynen M. Dadamio J. Van den Velde S. De Smit M. Dekeyser C. Van Tornout M.. (2009). Characteristics of 2000 patients who visited a halitosis clinic. J. Clin. periodontol. 36 (11), 970–975. doi: 10.1111/j.1600-051X.2009.01478.x 19811581

[B178] Rad A. Abbasi A. Javadi A. Pourjafar H. Javadi M. Khaleghi M. (2020a). Comparing the microbial quality of traditional and industrial yoghurts. Biointerface. Res. Appl. Chem. 10 (4), 6020–6025. doi: 10.33263/BRIAC104.020025

[B179] Rad A. H. Aghebati-Maleki L. Kafil H. S. Abbasi A. (2021a). Molecular mechanisms of postbiotics in colorectal cancer prevention and treatment. Crit. Rev. Food Sci. Nutr. 61 (11), 1787–1803. doi: 10.1080/10408398.2020.1765310 32410512

[B180] Rad A. H. Aghebati-Maleki L. Kafil H. S. Gilani N. Abbasi A. Khani N. (2021b). Postbiotics, as dynamic biomolecules, and their promising role in promoting food safety. Biointerface. Res. Appl. Chem. 11 (6), 14529–14544. doi: 10.33263/BRIAC116.1452914544

[B181] Rad A. H. Maleki L. A. Kafil H. S. Zavoshti H. F. Abbasi A. (2020b). Postbiotics as novel health-promoting ingredients in functional foods. Health Promotion. Perspect. 10 (1), 3–4. doi: 10.15171/hpp.2020.02 PMC7036208 32104650

[B182] Rams T. E. Degener J. E. Van Winkelhoff A. J. (2014). Antibiotic resistance in human chronic periodontitis microbiota. J. periodontol. 85 (1), 160–169. doi: 10.1902/jop.2013.130142 23688097

[B183] Reddy R. S. Swapna L. Ramesh T. Singh T. R. Vijayalaxmi N. Lavanya R. (2011). Bacteria in oral health-probiotics and prebiotics a review. Int. J. Biol. Med. Res. 2 (4), 1226–1233.

[B184] Relman D. A. Falkow S. (2001). The meaning and impact of the human genome sequence for microbiology. Trends Microbiol. 9 (5), 206–208. doi: 10.1016/S0966-842X(01)02041-8 11336835

[B185] Renuka P. Pushpanjali K. Sangeetha R. (2013). Review on “Influence of host genes on dental caries”. IOSR. J. Dent. Med. Sci. 4 (3), 86–92. doi: 10.9790/0853-0438692

[B186] Roberts F. A. Darveau R. P. (2002). Beneficial bacteria of the periodontium. Periodontol. 2000. 30 (1), 40–50. doi: 10.1034/j.1600-0757.2002.03004.x 12236894

[B187] Rodriguez E. Arques J. Rodriguez R. Nunez M. Medina M. (2003). Reuterin production by lactobacilli isolated from pig faeces and evaluation of probiotic traits. Lett. Appl. Microbiol. 37 (3), 259–263. doi: 10.1046/j.1472-765X.2003.01390.x 12904230

[B188] Saha S. Tomaro-Duchesneau C. Tabrizian M. Prakash S. (2012). Probiotics as oral health biotherapeutics. Expert Opin. Biol. Ther. 12 (9), 1207–1220. doi: 10.1517/14712598.2012.693474 22690730

[B189] Sales-Campos H. Soares S. C. Oliveira C. J. F. (2019). An introduction of the role of probiotics in human infections and autoimmune diseases. Crit. Rev. Microbiol. 45 (4), 413–432. doi: 10.1080/1040841X.2019.1621261 31157574

[B190] Sanders E. (1969). Bacterial interference: I. its occurrence among the respiratory tract flora and characterization of inhibition of group a streptococci by viridans streptococci. J. Infect. Dis. 120 (6), 698–707. 4986937 10.1093/infdis/120.6.698

[B191] Sartor R. (2016). The potential mechanisms of action of rifaximin in the management of inflammatory bowel diseases. Alimen. Pharmacol. Ther. 43, 27–36. doi: 10.1111/apt.13436 26618923

[B192] Sawada D. Sugawara T. Ishida Y. Aihara K. Aoki Y. Takehara I.. (2016). Effect of continuous ingestion of a beverage prepared with lactobacillus gasseri CP2305 inactivated by heat treatment on the regulation of intestinal function. Food Res. Int. 79, 33–39. doi: 10.1016/j.foodres.2015.11.032

[B193] Selwitz R. H. Ismail A. I. Pitts N. B. (2007). Dental caries. Lancet 369 (9555), 51–59. doi: 10.1016/S0140-6736(07)60031-2 17208642

[B194] Shakovetz N. Borutta A. Kneist S. (2013). Probiotica in caries prevention for preschool children. Oralprophylaxe. und. Kinderzahnheilkunde. 35 (3), 120–126.

[B195] Shimada A. Noda M. Matoba Y. Kumagai T. Kozai K. Sugiyama M. (2015). Oral lactic acid bacteria related to the occurrence and/or progression of dental caries in Japanese preschool children. Biosci. Microbiota. Food Health 32 (2), 29–36. doi: 10.12938/bmfh.2014-015 PMC4405395 25918670

[B196] Sidhu G. K. Mantha S. Murthi S. Sura H. Kadaru P. Jangra J. K. (2015). Evaluation of lactobacillus and streptococcus mutans by addition of probiotics in the form of curd in the diet. J. Int. Oral. Health.: JIOH. 7 (7), 85. PMC4513783 26229377

[B197] Silva Mendez L. Allaker R. Hardie J. Benjamin N. (1999). Antimicrobial effect of acidified nitrite on cariogenic bacteria. Oral. Microbiol. Immunol. 14 (6), 391–392. doi: 10.1034/j.1399-302X.1999.140612.x 10895698

[B198] Simon L. (2007). The role of streptococcus mutans and oral ecology in the formation of dental caries.

[B199] Singh R. Damle S. G. Chawla A. (2011). Salivary mutans streptococci and lactobacilli modulations in young children on consumption of probiotic ice-cream containing bifidobacterium lactis Bb12 and lactobacillus acidophilus La5. Acta Odontol. Scand. 69 (6), 389–394. doi: 10.3109/00016357.2011.572289 21466258

[B200] Slawik S. Staufenbiel I. Schilke R. Nicksch S. Weinspach K. Stiesch M.. (2011). Probiotics affect the clinical inflammatory parameters of experimental gingivitis in humans. Eur. J. Clin. Nutr. 65 (7), 857–863. doi: 10.1038/ejcn.2011.45 21448219

[B201] Slots J. Rams T. E. (1991). New views on periodontal microbiota in special patient categories. J. Clin. periodontol. 18 (6), 411–420. doi: 10.1111/j.1600-051X.1991.tb02309.x 1890221

[B202] Socransky S. S. Haffajee A. D. (1992). The bacterial etiology of destructive periodontal disease: current concepts. J. periodontol. 63, 322–331. doi: 10.1902/jop.1992.63.4s.322 1573546

[B203] Sookkhee S. Chulasiri M. Prachyabrued W. (2001). Lactic acid bacteria from healthy oral cavity of Thai volunteers: inhibition of oral pathogens. J. Appl. Microbiol. 90 (2), 172–179. doi: 10.1046/j.1365-2672.2001.01229.x 11168719

[B204] SrivaStava S. Saha S. Kumari M. Mohd S. (2016). Effect of probiotic curd on salivary pH and streptococcus mutans: a double blind parallel randomized controlled trial. J. Clin. Diagn. Res.: JCDR 10 (2), ZC13. doi: 10.7860/JCDR/2016/15530.7178 PMC4800643 27042577

[B205] Stamatova I. Kari K. Vladimirov S. Meurman J. H. (2009). *In vitro* evaluation of yoghurt starter lactobacilli and lactobacillus rhamnosus GG adhesion to saliva-coated surfaces. Oral. Microbiol. Immunol. 24 (3), 218–223. doi: 10.1111/j.1399-302X.2008.00498.x 19416451

[B206] Stamatova I. Meurman J. H. (2009). Probiotics: health benefits in the mouth. Am. J. Dentistry. 22 (6), 329. 20178208

[B207] Stecksén-Blicks C. Sjöström I. Twetman S. (2009). Effect of long-term consumption of milk supplemented with probiotic lactobacilli and fluoride on dental caries and general health in preschool children: a cluster-randomized study. Caries. Res. 43 (5), 374–381. doi: 10.1159/000235581 19690413

[B208] Stensson M. Koch G. Coric S. Abrahamsson T. Jenmalm M. Birkhed D.. (2014). Oral administration of lactobacillus reuteri during the first year of life reduces caries prevalence in the primary dentition at 9 years of age. Caries. Res. 48 (2), 111–117. doi: 10.1159/000354412 24296746

[B209] Strahinic I. Busarcevic M. Pavlica D. Milasin J. Golic N. Topisirovic L. (2007). Molecular and biochemical characterizations of human oral lactobacilli as putative probiotic candidates. Oral. Microbiol. Immunol. 22 (2), 111–117. doi: 10.1111/j.1399-302X.2007.00331.x 17311634

[B210] Sullivan Å. Nord C. (2005). Probiotics and gastrointestinal diseases. J. Internal Med. 257 (1), 78–92. doi: 10.1111/j.1365-2796.2004.01410.x 15606379

[B211] Šušković J. Kos B. Beganović J. Leboš Pavunc A. Habjanič K. Matošić S. (2010). Antimicrobial activity–the most important property of probiotic and starter lactic acid bacteria. Food Technol. Biotechnol. 48 (3), 296–307.

[B212] Sutula J. Coulthwaite L. A. Thomas L. V. Verran J. (2013). The effect of a commercial probiotic drink containing lactobacillus casei strain shirota on oral health in healthy dentate people. Microbial. Ecol. Health Dis. 24 (1), 21003. doi: 10.3402/mehd.v24i0.21003 PMC3813825 24179468

[B213] Suzuki N. Higuchi T. Nakajima M. Fujimoto A. Morita H. Yoneda M.. (2016). Inhibitory effect of enterococcus faecium WB2000 on volatile sulfur compound production by porphyromonas gingivalis. Int. J. Dentistry. 2016. doi: 10.1155/2016/8241681 PMC5075313 27799940

[B214] Szkaradkiewicz A. K. Stopa J. Karpiński T. M. (2014). Effect of oral administration involving a probiotic strain of lactobacillus reuteri on pro-inflammatory cytokine response in patients with chronic periodontitis. Archivum. Immunol. Ther. Experimentalis. 62 (6), 495–500. doi: 10.1007/s00005-014-0277-y PMC4244533 24509697

[B215] Taipale T. Pienihäkkinen K. Alanen P. Jokela J. Söderling E. (2013). Administration of bifidobacteriumanimalis subsp. lactis BB-12 in early childhood: a post-trial effect on caries occurrence at four years of age. Caries. Res. 47 (5), 364–372. doi: 10.1159/000348424 23571819

[B216] Taipale T. Pienihäkkinen K. Salminen S. Jokela J. Söderling E. (2012). Bifidobacteriumanimalis subsp. lactis BB-12 administration in early childhood: a randomized clinical trial of effects on oral colonization by mutans streptococci and the probiotic. Caries. Res. 46 (1), 69–77. doi: 10.1159/000335567 22327347

[B217] Takahashi N. (2015). Oral microbiome metabolism: from “who are they?” to “what are they doing”? J. Dental Res. 94 (12), 1628–1637. doi: 10.1177/0022034515606045 26377570

[B218] Takahashi N. Nyvad B. (2011). The role of bacteria in the caries process: ecological perspectives. J. Dental Res. 90 (3), 294–303. doi: 10.1177/0022034510379602 20924061

[B219] Talarico T. Casas I. Chung T. C. Dobrogosz W. (1988). Production and isolation of reuterin, a growth inhibitor produced by lactobacillus reuteri. Antimicrob. Agents Chemother. 32 (12), 1854–1858. doi: 10.1128/AAC.32.12.1854 3245697 PMC176032

[B220] Tanner A. Kressirer C. Rothmiller S. Johansson I. Chalmers N. (2018). The caries microbiome: implications for reversing dysbiosis. Adv. Dental Res. 29 (1), 78–85. doi: 10.1177/0022034517736496 29355414

[B221] Tanner A. Mathney J. Kent R. Chalmers N. Hughes C. Loo C.. (2011). Cultivable anaerobic microbiota of severe early childhood caries. J. Clin. Microbiol. 49 (4), 1464–1474. doi: 10.1128/JCM.02427-10 21289150 PMC3122858

[B222] Tanner A. Milgrom P. Kent R. Jr. Mokeem S. Page R. Liao S.. (2002). Similarity of the oral microbiota of pre-school children with that of their caregivers in a population-based study. Oral. Microbiol. Immunol. 17 (6), 379–387. doi: 10.1034/j.1399-302X.2002.170608.x 12485330

[B223] Tanzer J. M. Livingston J. Thompson A. M. (2001). The microbiology of primary dental caries in humans. J. Dental Educ. 65 (10), 1028–1037. doi: 10.1002/j.0022-0337.2001.65.10.tb03446.x 11699974

[B224] Teanpaisan R. Piwat S. (2014). Lactobacillus paracasei SD1, a novel probiotic, reduces mutans streptococci in human volunteers: a randomized placebo-controlled trial. Clin. Oral. Invest. 18 (3), 857–862. doi: 10.1007/s00784-013-1057-5 23892501

[B225] Teanpaisan R. Piwat S. Tianviwat S. Sophatha B. Kampoo T. (2015). Effect of long-term consumption of lactobacillus paracasei SD1 on reducing mutans streptococci and caries risk: a randomized placebo-controlled trial. Dentistry. J. 3 (2), 43–54. doi: 10.3390/dj3020043 PMC5851198 29567924

[B226] Teughels W. Durukan A. Ozcelik O. Pauwels M. Quirynen M. Haytac M. C. (2013). Clinical and microbiological effects of lactobacillus reuteri probiotics in the treatment of chronic periodontitis: a randomized placebo-controlled study. J. Clin. periodontol. 40 (11), 1025–1035. doi: 10.1111/jcpe.12155 24164569 PMC3908359

[B227] Teughels W. Kinder Haake S. Sliepen I. Pauwels M. Van Eldere J. Cassiman J.-J.. (2007). Bacteria interfere with a. actinomycetemcomitans colonization. J. Dental Res. 86 (7), 611–617. doi: 10.1177/154405910708600706 17586706

[B228] Toiviainen A. Jalasvuori H. Lahti E. Gursoy U. Salminen S. Fontana M.. (2015). Impact of orally administered lozenges with lactobacillus rhamnosus GG and bifidobacterium animalis subsp. lactis BB-12 on the number of salivary mutans streptococci, amount of plaque, gingival inflammation and the oral microbiome in healthy adults. Clin. Oral. Invest. 19 (1), 77–83. doi: 10.1007/s00784-014-1221-6 PMC4286650 24638207

[B229] Trüper H. G. De’Clari L. (1997). Taxonomic note: necessary correction of specific epithets formed as substantives (nouns)”in apposition”. Int. J. Syst. Evol. Microbiol. 47 (3), 908–909. doi: 10.1099/00207713-47-3-908

[B230] Twetman S. (2012). Are we ready for caries prevention through bacteriotherapy? Braz. Oral. Res. 26, 64–70. doi: 10.1590/S1806-83242012000700010 23318746

[B231] Twetman S. Derawi B. Keller M. Ekstrand K. Yucel-Lindberg T. Stecksen-Blicks C. (2009b). Short-term effect of chewing gums containing probiotic lactobacillus reuteri on the levels of inflammatory mediators in gingival crevicular fluid. Acta Odontol. Scand. 67 (1), 19–24. doi: 10.1080/00016350802516170 18985460

[B232] Twetman S. Keller M. (2012). Probiotics for caries prevention and control. Adv. Dental Res. 24 (2), 98–102. doi: 10.1177/0022034512449465 22899689

[B233] Twetman L. Larsen U. Fiehn N.-E. Stecksén-Blicks C. Twetman S. (2009a). Coaggregation between probiotic bacteria and caries-associated strains: an *in vitro* study. Acta Odontol. Scand. 67 (5), 284–288. doi: 10.1080/00016350902984237 19479452

[B234] Valdez R. M. A. Dos Santos V. R. Caiaffa K. S. Danelon M. Arthur R. A. de Cássia Negrini T.. (2016). Comparative *in vitro* investigation of the cariogenic potential of bifidobacteria. Arch. Oral. Biol. 71, 97–103. doi: 10.1016/j.archoralbio.2016.07.005 27475723

[B235] Van den Broek A. M. Feenstra L. de Baat C. (2007). A review of the current literature on aetiology and measurement methods of halitosis. J. Dentistry 35 (8), 627–635. doi: 10.1016/j.jdent.2007.04.009 17555859

[B236] Van Houte J. (1994). Role of micro-organisms in caries etiology. J. Dental Res. 73 (3), 672–681. doi: 10.1177/00220345940730031301 8163737

[B237] Villavicencio J. Villegas L. M. Arango M. C. Arias S. Triana F. (2018). Effects of a food enriched with probiotics on streptococcus mutans and lactobacillus spp. salivary counts in preschool children: a cluster randomized trial. J. Appl. Oral. Sci. 26. doi: 10.1590/1678-7757-2017-0318 PMC5958937 29768525

[B238] Vivekananda M. Vandana K. Bhat K. (2010). Effect of the probiotic lactobacilli reuteri (Prodentis) in the management of periodontal disease: a preliminary randomized clinical trial. J. Oral. Microbiol. 2 (1), 5344. doi: 10.3402/jom.v2i0.5344 PMC3084569 21523225

[B239] Volozhin A. Il'in V. IuM M. Sidorenko A. Istranov L. Tsarev V.. (2004). Development and use of periodontal dressing of collagen and lactobacillus casei 37 cell suspension in combined treatment of periodontal disease of inflammatory origin (a microbiological study). Stomatologiia 83 (6), 6–8. 15602477

[B240] Walker R. Buckley M. (2006). Probiotic microbes: the scientific basis. 32687280

[B241] Wattanarat O. Makeudom A. Sastraruji T. Piwat S. Tianviwat S. Teanpaisan R.. (2015). Enhancement of salivary human neutrophil peptide 1–3 levels by probiotic supplementation. BMC Oral. Health 15 (1), 1–11. doi: 10.1186/s12903-015-0003-0 25884192 PMC4327807

[B242] Werneck R. Mira M. Trevilatto P. (2010). A critical review: an overview of genetic influence on dental caries. Oral. Dis. 16 (7), 613–623. doi: 10.1111/j.1601-0825.2010.01675.x 20846151

[B243] Wescombe P. A. Heng N. C. Burton J. P. Tagg J. R. (2010). Something old and something new: an update on the amazing repertoire of bacteriocins produced by streptococcus salivarius. Probiot. Antimicrob. Proteins 2 (1), 37–45. doi: 10.1007/s12602-009-9026-7 26780899

[B244] Wilson M. (2005). Microbial inhabitants of humans: Their ecology and role in health and disease (Cambridge University Press).

[B245] Wolff L. Dahlén G. Aeppli D. (1994). Bacteria as risk markers for periodontitis. J. Periodontol. 65, 498–510. doi: 10.1902/jop.1994.65.5s.498 8046566

[B246] Yadav M. Poornima P. Roshan N. Prachi N. Veena M. Neena I. (2014). Evaluation of probiotic milk on salivary mutans streptococci count: an *in vivo* microbiological study. J. Clin. Pediatr. Dentistry 39 (1), 23–26. doi: 10.17796/jcpd.39.1.u433n8w245511781 25631721

[B247] Yaegaki K. Coil J. M. (2000). Examination, classification, and treatment of halitosis; clinical perspectives. Journal-canadian. Dental Assoc. 66 (5), 257–261. 10833869

[B248] Yasui H. Shida K. Matsuzaki T. Yokokura T. (1999). Immunomodulatory function of lactic acid bacteria. Lactic. Acid Bacteria.: Genet. Metab. Appl., 383–389. doi: 10.1007/978-94-017-2027-4_24 10532394

[B249] Yoon B. K. Jackman J. A. Valle-González E. R. Cho N.-J. (2018). Antibacterial free fatty acids and monoglycerides: biological activities, experimental testing, and therapeutic applications. Int. J. Mol. Sci. 19 (4), 1114. doi: 10.3390/ijms19041114 29642500 PMC5979495

[B250] Yousuf A. Nagaraj A. Ganta S. Sidiq M. Pareek S. Vishnani P.. (2015). Comparative evaluation of commercially available freeze dried powdered probiotics on mutans streptococci count: a randomized, double blind, clinical study. J. Dentistry. (Tehran. Iran). 12 (10), 729. PMC4888159 27252756

[B251] Zainab J. Raghad F. Yasameen A. (2016). Correlation between caries related microorganisms in the dental plaque and saliva with dental caries level in the upper and lower jaws in 5-9 years old children in baghdad city. J. Bagh. Coll. Dentistry. 28 (3), 132–136.

[B252] Zaura E. Keijser B. J. Huse S. M. Crielaard W. (2009). Defining the healthy" core microbiome" of oral microbial communities. BMC Microbiol. 9 (1), 1–12. doi: 10.1186/1471-2180-9-259 20003481 PMC2805672

[B253] Zhai J.-J. Zou J. Lu L.-Y. (2009). Distribution of bifidobacterium in oral cavities of children and the relations with caries. Hua. xi. kou. Qiang. yi. xue. za. zhi=. Huaxi. Kouqiang. Yixue. Zazhi=. West. China J. Stomatol. 27 (6), 618–621. 20077895

[B254] Żółkiewicz J. Marzec A. Ruszczyński M. Feleszko W. (2020). Postbiotics–a step beyond pre-and probiotics. Nutrients 12 (8), 2189. doi: 10.3390/nu12082189 32717965 PMC7468815

